# Nutrient-depended metabolic switching during batch cultivation of *Streptomyces coelicolor* explored with absolute quantitative mass spectrometry-based metabolite profiling

**DOI:** 10.1007/s13205-022-03146-x

**Published:** 2022-02-26

**Authors:** Kanhaiya Kumar, Per Bruheim

**Affiliations:** Department of Biotechnology and Food Science, Norwegian University of Science and Technology (NTNU), 7491 Trondheim, Norway

**Keywords:** Metabolomics, Targeted metabolite profiling, LC–MS/MS, Secondary metabolism, Antibiotics, Bioreactor

## Abstract

The well-known secondary metabolite-producing bacterium *Streptomyces coelicolor* is a natural choice for the development of super-hosts optimized for the heterologous expression of antibiotic biosynthetic gene clusters (BGCs). In this study, we used *S. coelicolor* M145 and its derivative strain M1146 where all active BGCs have been deleted and generated high-resolution quantitative time series metabolite profiles under two cultivation conditions (phosphate and nitrogen limitation to cease growth and trigger secondary metabolism). Five targeted LC–MS/MS-based methods were used to quantify intracellular primary metabolites covering phosphorylated metabolites, amino acids, organic acids, (deoxy) nucleoside/sugar phosphates, Nicotinamide adenine dinucleotide (NAD), and Coenzyme A (CoA*)*. The nitrogen limitation resulted in a sharp decline in respiration and an immediate drop in the cell mass concentration. Intracellularly, a reduction in the level of the metabolites next to α-ketoglutarate in the tricarboxylic acid cycle and a decrease in the NADH pool were among the most prominent adaptation to this nutrient limitation. Phosphate limitation evoked a different adaptation of the metabolite pools as most of the phosphorylated metabolite pools except 6-phosphogluconic acid (6PG) pool were downregulated. ^13^C-isotope-labeling experiments revealed the simultaneous activity of both glycolysis and gluconeogenesis during the co-utilization of glucose and glutamate. The *S. coelicolor* M1146 strain had similar time-series metabolite profile dynamics as the parent M145 strain, except for a visibly increased 6PG pool in the stationary phase. In general, the nutrient limitation had a larger effect on the metabolite pool levels than the absence of secondary metabolite production in M1146. This study provides new insight into the primary carbon metabolism and its link to the secondary metabolism which is needed for further optimization of both super-host genotype and cultivation conditions.

**Supplementary Information:**

The online version contains supplementary material available at 10.1007/s13205-022-03146-x.

## Introduction

Antibiotic resistance is dramatically increasing requiring enhanced efforts to search for new antibiotics to combat the emerging clinical crisis (Hawkey and Jones 2009; Hwang et al. 2014). The rediscovery of the same bioactive biomolecules is one of the main bottlenecks undermining global efforts*. Actinobacteria* contribute to over half of the identified bacterial bioactive molecules and among these, around 70% of the antibiotics are produced by the *Streptomyces* genus (Mahajan and Balachandran 2012; Ranjani et al. 2016). *Streptomyces coelicolor* A3(2) is a well-studied model system producing different types of secondary metabolites (antibiotics, pigments) e.g. actinorhodin, undecylprodigiosin, calcium-dependent antibiotic (CDA) (Rioseras et al. 2014; Bystrykh et al. 1996). It was the first *Streptomyces* sp. to be fully genome sequenced and contains almost thirty cryptic tentative secondary metabolite gene clusters (BGCs) (Bentley et al. 2002). The genetic toolbox is quite advanced which has made it the most frequently used model strain to investigate the regulation of antibiotic biosynthesis (Kieser et al. 2000). Thus, *S. coelicolor* is a natural choice for the development of optimized super-hosts for the heterologous expression of BGCs from different native sources to produce new bioactive compounds.

The central carbon metabolites (CCMs) carry most of the metabolic flux and provide precursors, reductive power, and energy used for biomass generation and secondary metabolite synthesis and their analysis can give real-time status of phenotype (Coze et al. 2013; Kumar et al. 2021; Rost et al. 2020a; Kumar and Bruheim 2021a, b). Therefore, information about the intracellular metabolite concentrations provided by metabolome analysis will further enhance our understanding of the genotype–phenotype interactions both by direct single interpretation of metabolomics data but also when integrated with multi omics-data such as genomics (Xu et al. 2017; Nguyen et al. 2012), transcriptomics (Kim and Kim 2016; Yague et al. 2014; Nieselt et al. 2010) and proteomics (Thomas et al. 2012). Mathematical modeling, such as genome-scale metabolic models will also become important in this workflow (Amara et al. 2018; Borodina et al. 2005; Sulheim et al. 2020).

The success of comprehensive and absolute quantitative metabolite profiling depends upon robust and reproducible cultivation conditions combined with optimized workflow consisting of several steps such as sampling methods, sample processing, and the efficiency of the LC–MS/MS system to generate high-resolution metabolite data (Villas-Boas and Bruheim 2007). The filamentous growth of *Streptomyces* makes it challenging for homogeneous and consistent sampling (Yague et al. 2010). Previously, cultivation conditions of *S.coelicolor* A3(2) were improved to obtain dispersed and reproducible growth by subjecting it to a high agitation rate causing high shear stress (Wentzel et al. 2012a). In the same study, a media was reported consisting of two carbon sources: D-glucose and L-glutamate; that at optimized concentrations delay the onset of secondary metabolism and provide enough biomass for sampling during the distinct growth, transition and antibiotic production phases. Several studies have reported metabolome data based on the analysis of secondary metabolites (Cihak et al. 2017; Goodwin et al. 2015; Lee et al. 2015; Morgenstern et al. 2015; Traxler et al. 2013), but only a few studies are reported for understanding the physiology of *S.coelicolor* by diving into comprehensive coverage of primary metabolites (Nitta et al. 2020; Wentzel et al. 2012b). Comparing the metabolite data sets from different laboratories is also challenging because they are generated using different continuously improved analytical methodologies, under different cultivation set-ups and physiological conditions. Most data is also only reported as abundances and compared on a relative scale.

Primary metabolites have both different physicochemical properties and roles, based on which they are classified into different metabolite classes [i.e. amino acids, organic acids, phosphorylated metabolites (deoxy) nucleoside/sugar phosphates, and cofactors like Nicotinamide adenine dinucleotide (NAD) and Coenzyme A (CoA) metabolites]). This is the reason to adopt several metabolite class-specific sampling and analytical protocols for comprehensive and complete quantitative coverage of the CCMs. We have developed a set of LC–MS/MS methods for the absolute quantitative metabolite profiling including the ^13^C-isotope dilution approach for enhanced analytical accuracy (Supplementary Fig. S1) (Bartosova et al. 2021; Kumar et al. 2021; Rost et al. 2020a; Kumar and Bruheim 2021b). The current study aimed to apply these methods for the quantification of CCMs of *S. coelicolor* M145 during the growth and antibiotic production phases in two nutrient-limited conditions (L-glutamate and phosphate limited). *S coelicolor* M145 is a plasmid cured derivative strain of the wild type strain A3(2) (Kieser et al. 2000) and a reference strain of a series of new derivative strains (Gomez-Escribano and Bibb 2011). Recently, we have published a comprehensive coverage of primary metabolome data by comparing super host strain *S. coelicolor* M1152 and its derivative *S. coelicolor* M1581 heterologously expressing a chloramphenicol BGC (Kumar and Bruheim 2021b). The current study was designed to further decipher the distribution and participation of carbon from two substrates (D-glucose and L-glutamate) in primary metabolic pathways and their role in the growth and support of antibiotic biosynthesis. The M145 reference strain and the derivative strain *S. coelicolor* M1146 where the four native BGCs being expressed (actinorhodin, undecylprodigiosin, calcium-dependent antibiotics, methylenomycin) have been inactivated were selected for this study as this mutant strain provides a cleaner background for heterologous expression of new and potentially bioactive secondary metabolites. Importantly, this is probably the first report on LC–MS/MS-based absolute quantification of intracellular concentrations of NAD, NADH, NADP, and NADPH during growth, and secondary metabolite production phases. From a super-host development perspective, it is especially critical to verify that sufficient NADPH supplies are available throughout all cultivation stages.

## Materials and methods

### Strains and cultivation conditions

Two strains of *Streptomyces coelicolor* were used in this study: *S. coelicolor* M145 and *S. coelicolor* M1146. The protocols for spore preparation, pre-germination, media preparation, and setting up the experiments were adopted from earlier reports (Wentzel et al. 2012a; Kumar and Bruheim 2021b). The cultivations were conducted in 3 L bioreactors (Eppendorf Bioflo 320) with an initial working volume of 1.8 L and the inoculum was pre-germinated spores having ~ 1 × 10^9^ CFU L^−1^. The study was conducted in phosphate (SSBM-P) and nitrogen (L-glutamate) limited (SSBM-E) media at 30 °C, pH 7.0. SSBM media consisted of two carbon sources: D-glucose and L-glutamate. SSBM-P media consisted of D-glucose (40 g L^−1^), Sodium L-glutamate monohydrate (61.1 g L^−1^), Phosphate (4.6 mM), Magnesium sulfate (2.0 mM), and previously reported minimal medium trace element solution (Wentzel et al. 2012a). SSBM-E media had a similar composition as SSBM-P media except for a reduced amount of Sodium L-glutamate monohydrate (16.6 g L^−1^), and an increased amount of phosphate (9.2 mM). The ^13^C isotope labeling experiments were conducted at 30 °C, pH 7.0, in customized mini bioreactors (working volume of 200 mL) where the agitation was provided using a magnetic stirrer. The SSBM-P media used in the study had a similar composition as stated above except the D-glucose was ^13^C labeled at all carbon positions (U-^13^C6, 99%, Cambridge Isotope Laboratories, Inc.) and its concentration was reduced to 20 g L^−1^. Time-series sampling was analyzed for better understating and the direct interpretation of dynamic ^13^C labeling patterns (Buescher et al. 2015).

### Quantification of exometabolites

The fermentation broth was sampled into 15 mL centrifuge tubes and biomass pelleted using centrifugation at 4500 × g, 4 °C and 5 min. The pellets were washed three times with NaCl solution (9 g L^−1^) followed by three times with ultrapure water using centrifugation. Dry cell weight (DCW) was quantified by transferring biomass pellets into a pre-dried and pre-weighed aluminum pan and dried at 110 °C till constant weight was achieved.

The supernatant of the fermentation broth was used for the analysis of D-glucose and excreted organic acids using high-performance liquid chromatography (HPLC). Before analysis, the supernatant samples were filtered using syringe filters with a 0.2 μm polyethersulfone membrane (VWR). The Waters Alliance HPLC was equipped with a refractive index (RI) and a UV/VIS detector to measure the carbohydrates and organic acids, respectively. The Hi-Plex column of dimension 300 × 7.7 mm was used to separate the sugars and organic acids. The column and the sample compartment were set at 45 °C and 10 °C, respectively. The mobile phase was 0.05 M H_2_SO_4_ in ultrapure water at the flow rate of 0.8 mL min^−1^.

The extracellular L-glutamate was analyzed separately using LC–MS/MS following a similar protocol (described later) as for the intracellular amino acid analysis method except using a shorter C18 column (1.7 µm, 2.1 × 75 mm) to reduce the analysis run time.

The intracellular (actinorhodin, *Act*) and extracellular (γ *Act)* blue pigment were extracted from biomass pellets and supernatants, respectively. The protocol was adopted from the previous report (Coze et al. 2013). The *Act* was analyzed by resuspending the pellet in 1.0 mL KOH (1.0 M), and with thorough mixing for 20 min at 4 °C and centrifuged at 20,817 × g, 4 °C, and 5 min. After this step, *Act* and γ *Act* were extracted following similar steps. The supernatant was collected and 0.5 mL HCl (3.0 M) was added to precipitate the *Act*. The *Act* was pelleted by incubating the sample at 4 °C for 15 min, followed by centrifugation at 20,817 × g, 4 °C for 5 min. The pellet was resuspended in 1.0 mL KOH (1.0 M) and the absorbance of the samples was measured at 644 nm using a plate reader. The *Act* concentration was calculated using the molar extinction coefficient of 25,320 L mol^−1^ cm^−1^ for pure *Act* in 1.0 M KOH.

The extraction of intracellular red pigment (Undecylprodigiosin, *Red*) was adopted from the previous report (Sheng-wan Tsao et al. 1985). The biomass pellet obtained from 1.0 mL fermentation broth was resuspended in 1.0 mL methanol, and with thorough mixing for 30 min at 4 °C, and centrifuged at 20,817 × g, 4 °C, and 5 min. The supernatant was collected and 0.5 mL HCl was added, and the sample was incubated at room temperature for 5 min and followed by centrifugation at 20,817 × g for 5 min at room temperature. The absorbance of the samples was measured at 530 nm using a plate reader. The *Red* concentration was calculated using the molar extinction coefficient of 100,500 L mol^−1^ cm^−1^ for the pure *Red* in an equal volume of methanol/HCl (1 M).

### Quantification of endometabolites

#### Sampling and sample preparation

Time-series sampling for intracellular metabolite profiling was conducted in the exponential and the nutrient depletion phase. The fermentation cell suspension was pipetted from the bioreactors and immediately filtered using a Durapore^®^ 0.65 μm polyvinylidene fluoride filter (Merck) through a funnel connected to a vacuum (200 bar), and rinsed with 10 mL, ice-cooled 2.3% NaCl solution followed by 10 mL ice-cooled, ultrapure water. The filter containing the biomass was transferred to 13 mL ice-cooled 55:45 ACN: H_2_O solution in 50 mL tubes, followed by immediate quenching in liquid nitrogen, N_2_(*l*), and stored at -80 °C until further processing.

The intracellular metabolite from the samples was extracted following three times freezing and thawing and with an intermittent brief vertexing after each thawing. The thawing step was conducted at 0 °C in an ethanol bath and freezing was done using liquid nitrogen, N_2_(*l*). This was followed by centrifugation (4500 × g, 4 °C, 10 min) and aliquoting 3.5 mL of sample into three 15 mL tubes. The aliquoted samples were dried for ~ 24 h at − 105 °C and 12 Pa in a freeze dryer. The dried metabolites were re-constituted in 550 μL HPLC-grade water and centrifuged (4500 × g, 4 °C, 5 min) to remove large cell debris. The supernatant was transferred into 3-kDa-molecular-weight spin cut-off filters (516-0228, VWR) and centrifuged at 20,817 × g, 4 °C, 5 min to get the sample for further analysis.

#### Amino acids, organic acids, and phosphorylated metabolites analysis

For amino acids and organic acids, the samples were first derivatized before quantification using LC–MS/MS. The derivatization protocol and LC–MS/MS analysis applied for amino acids and organic acids were adopted as previously reported (Kumar et al. 2021). All phosphorylated metabolites and some tricarboxylic acid (TCA) cycle metabolites were quantified using capillary ion chromatography and tandem mass spectroscopy (CapIC-MS/MS) as described in previous reports (Kvitvang et al. 2014; Kumar et al. 2021; Stafsnes et al. 2018).

#### Nicotinamide adenine dinucleotide (NAD) and Coenzyme A (CoA) metabolites analysis

A different sampling protocol had to be developed for the quantitation of CoAs and NADs. First, pellets were collected by centrifugation at 4 °C, for one minute at a maximum speed: 4500 × g for 15 mL centrifuge tubes (for exponential phase samples with less biomass) and 20,817 × g for 2 mL centrifuge tubes (for stationary phase samples). Supernatants were discarded, and pellets were quickly quenched in N_2_(*l*) and stored at − 80 °C until further analysis. NAD and CoA metabolites extraction were optimized using *S. coelicolor* as a biological model system and then extracted following cold extraction and quantified as described previously (Bartosova et al. 2021; Kumar and Bruheim 2021a; Rost et al. 2020b). Cold extractions in 50 mM NH_4_Ac having a composition of acetonitrile:buffer:CH_3_OH (7:2:1) including 5% of a ^13^C-labelled biological extract (see below), at pH 9 and 5, respectively, for NADs and CoAs. The extracts were incubated at 4 °C for 3 min while agitating at 1500 rpm. ^13^C-labeled biological extract (*E.coli*, 10 mL, OD 2.3) was prepared similarly. Samples were centrifuged at 4 °C, 2 min, 20,817 × g for clearing cell debris. It was followed by transferring supernatants into 3-kDa-molecular-weight spin cut-off filters (516-0228, VWR) and centrifugation at 4 °C, 5 min, 20,817 × g. The standards used to prepare the calibration curves were prepared in a matrix containing 5%, ^13^C-labelled *E.coli* biological extract. Chromatographic separation was adopted from a previous report (Rost et al. 2020b) and performed using hydrophilic interaction liquid chromatography (HILIC) coupled with tandem mass spectroscopy. More about the development and assessment of the NAD and CoA quantitation can be found in Supplementary Figs. S2 and S3, respectively.

#### Data analysis and calculation

The specific growth rate, μ was calculated in the exponential phase, which is equal to the slope of ln[*x*] plotted as a function of time. The specific rates (specific D-glucose uptake rate, *q*_gluc_; specific L-glutamate uptake rate, *q*_glut_; specific *Act,* γ*Act* and *Red* production rate were *p*_Act_, *p*_*γ*Act_, *p*_red_, respectively**)** were calculated by dividing the volumetric rate by the average biomass between the two points. The carbon recovery percentage was measured between the first and the last point of analysis, which was calculated based on the amount of carbon (on a mole basis) recovered from the biomass, organic acids, CO_2,_ and products (pigments), and the amount of carbon supplied in the form of substrates. The molecular weight of biomass was assumed as 27.0 g dry weight/mol (Kumar and Bruheim 2021b). The respiratory quotient (RQ) was calculated from the ratio of CO_2_ evolution to oxygen consumption.

The MS data were processed using the TargetLynx 4.2 software. Integration was performed automatically but manually checked to ensure quality. All metabolite concentrations were calculated based on an individual linear standard calibration curve. All metabolites concentrations were normalized using biomass. All intracellular metabolites pools were reported as an average of two to three independent replicas and/or supported by another independent batch cultivation. The CCMs are visualized using the Omix software (Droste et al. 2011). The principal component analysis (PCA) scores plot was plotted for *S. coelicolor* M145 metabolite time series data in SSBM-E and SSBM-P media using MetaboAnalyst software (Xia et al. 2009). Missing values were replaced by feature mean and autoscaled before PCA.

The equation below was used to calculate the Energy charge (Atkinson 1977):

$$\mathrm{Energy charge ratio }\left(\mathrm{ECR}\right)= ([\mathrm{ATP}] + 0.5 \times [\mathrm{ADP}]) /([\mathrm{ATP}] + [\mathrm{ADP}] + [\mathrm{AMP}])$$

In the ^13^C labeled experiment, the relative summed fractional labeling (Rel SFL) of each metabolite was calculated (scale 0 to 100) as shown below, where I_n_ is the signal area of metabolite with n number of labeled carbon (Isotopologues) (Gombert et al. 2001).

$$\mathrm{Rel SFL }= (0\times {\mathrm{I }}_{0}+1\times {\mathrm{I }}_{1}+ 2 \times {\mathrm{I }}_{2}+ 3 \times {\mathrm{I }}_{3} + ...\mathrm{ n }\times {\mathrm{I }}_{n}) / ({{\mathrm{I }}_{0}+\mathrm{I }}_{1}+ {\mathrm{I }}_{2}+ {\mathrm{I }}_{3} + ... {\mathrm{I }}_{n})\times 100/n$$

## Results and discussion

In this study, we first present the cultivation data of the reference strain *S. coelicolor* M145 in the two different nutrients limited mediums (glutamate limited/N-lim: SSBM-E, and phosphate limited/P-lim: SSBM-P) and the derivative strain M1146 in SSBM-P conditions. Besides being an additional source of carbon, L-glutamate was the sole nitrogen source in the SSBM media. Therefore, the depletion of L-glutamate in the media corresponded to nitrogen limitation. No cultures are carbon limited at any state since glucose is in high abundance (never below 10 gL^−1^). Next, time series of primary metabolite pool profiles are presented and followed by the ^13^C-isotope labeling results to further enhance our understanding of the distribution and interaction of substrate carbon in the different central metabolic pathways. Finally, the metabolite pool composition of the derivative strain *S. coelicolor* M1146 is compared to the reference *S. coelicolor* M145 under P lim conditions.

### Growth kinetics of S. coelicolor M145

**Fig. 1:**
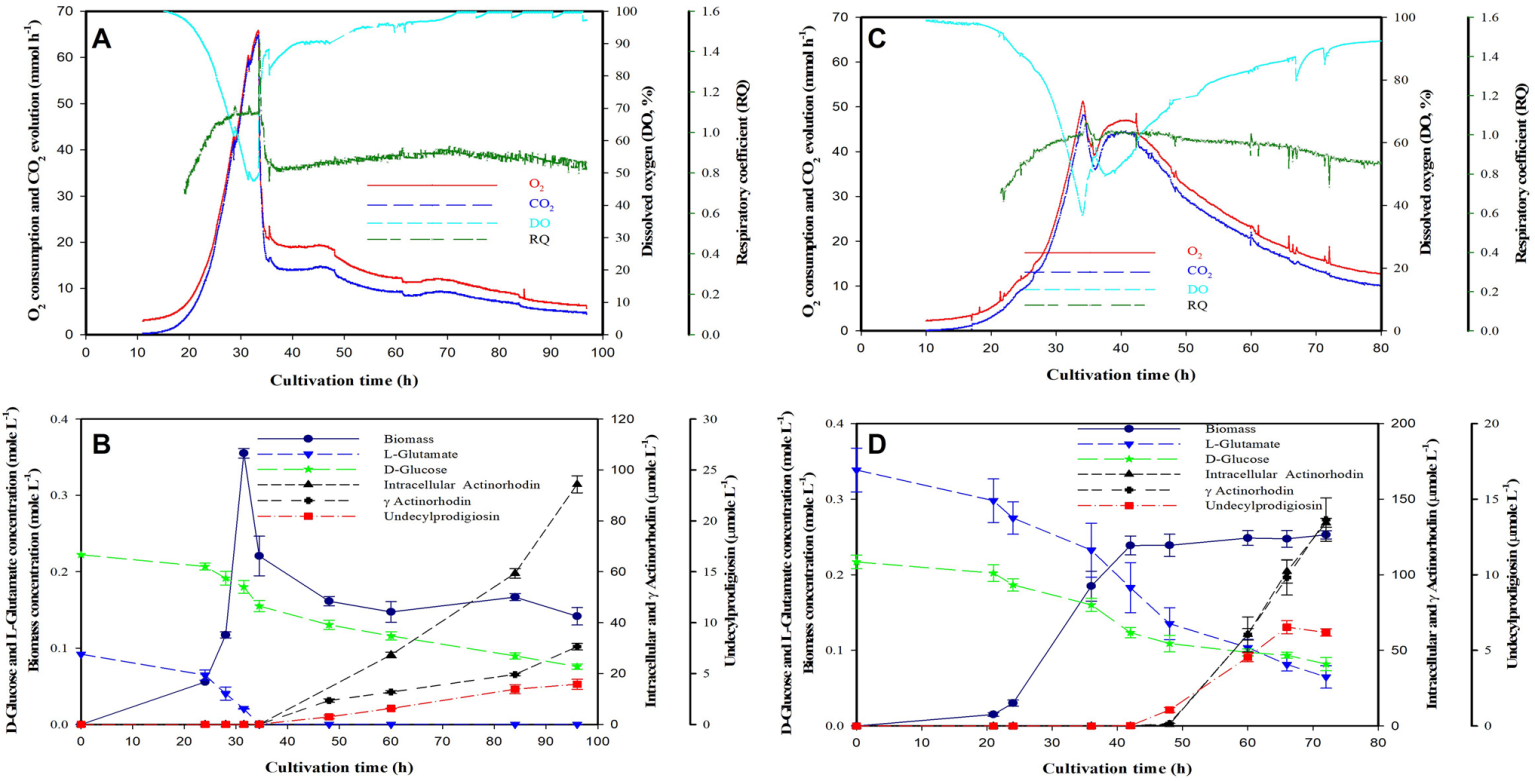
The online and offline cultivation data of *S. coelicolor* M145 in L-glutamate limited (**A** and **B**), and phosphate limited (**C** and **D**) media. The molecular weight of biomass is assumed as 27.0 g dry weight per mole. Offline data are the average of at least duplicate samples, with their respective standard deviation

L-glutamate/ N lim and P lim caused characteristic different CO_2_ evolution profiles (Fig. 1A and C). N lim resulted in a sharp decline in the CO_2_ evolution at the time of L-glutamate depletion, whereas a gradual decrease in the CO_2_ evolution was observed in P lim. The slope of the respiratory coefficient (RQ – ratio of CO_2_ production vs. O_2_ consumption) can be used to assess the respiratory state of the two cultivation conditions. In both cultivation conditions, RQ increased in the exponential phase till close to 1.1, followed by a sharp decline in N lim and a gradual decline in P lim (Fig. 1A and C). SSBM-E supported a slightly higher growth rate compared to the SSBM-P media (0.21 ± 0.04 vs. 0.16 ± 0.01 h^−1^) (Table 1), most likely due to the lower initial concentration of phosphate in P lim. There is a slight (statistically non-significant) trend that the specific L-glutamate uptake rate, *q*_glut_ was higher compared to the D-glucose uptake rate. The *q*_glut_ and *q*_gluc_ were higher in P lim compared to the N lim media even though the growth rate was lower. The depletion of either L-glutamate or phosphate at a defined time resulted in a switch to the secondary production phase from the growth phase with a large decrease in the substrate consumption rates. Three antibiotics/pigments were quantified: intracellular actinorhodin (*Act*), extracellular actinorhodin (*γ* *Act),* and undecylprodigiosin (*Red*), which were produced after nutrient limitation in both media (Fig. 1B and D). It was observed that the specific production rates (*p*_Act_, *p*_*γ*Act_, *p*_red_) of all three antibiotics/pigments were around 1.3, 2.0, 2.6 times higher in the P lim as compared to the N lim, respectively (Table 1). The M1146 strain cultivated in P lim conditions grew faster than M145 but the substrate consumption rates were comparable (Table 1 and Supplementary Fig. S7).

**Table 1:** The specific growth (m), substrate (q) and production (P) rates, and carbon recovery for *Streptomyces coelicolor* M145 in L-glutamate (N lim) and phosphate limited (P lim) mediums and M1146 in phosphate limited medium only for the two cultivation phase (growth—Expo, secondary metabolism—Stat)

	*S. coelicolor* M145				*S. coelicolor* M1146	
	N lim		P lim		P lim	
	Expo	Stat	Expo	Stat	Expo	Stat
µ (h^−1^)	0.21 ± 0.04	–	0.16 ± 0.01	–	0.25 ± 0.02	–
q_Glut_ (mol mol^−1^ DCW h^−1^)	0.03 ± 0.02	–	0.05 ± 0.04	0.02 ± 0.01	0.06 ± 0.04	0.01 ± 0.01
q_Glu_ (mol mol^−1^ DCW h^−1^)	0.02 ± 0.01	0.01 ± 0.01	0.04 ± 0.01	0.01 ± 0.00	0.06 ± 0.03	0.01 ± 0.01
P_Act_ (µmol mol^−1^ DCW h^−1^)	–	13.71 ± 1.14	–	17.50 ± 1.13	–	–
P_γAct_ (µmol mo^l−1^ DCW h^−1^)	–	8.24 ± 6.26	–	16.83 ± 2.17	–	–
P_Red_ (µmol mol^−1^ DCW h^−1^)	–	0.44 ± 0.06	–	1.12 ± 0.06	–	–
Carbon recovery %	74.4 ± 3.6		69.3 ± 8.5		78.4 ± 5.2	

### Mass spectrometric profiling of central intracellular metabolite pools

More than 80 metabolites, with almost complete coverage in the pentose phosphate pathway (PPP), glycolytic, deoxy-/nucleoside, and sugar phosphates, amino acids, organic acids, CoA, and NAD metabolite classes, were quantified by applying five different LC–MS/MS methods (Supplementary Fig. S1). The time-series metabolite analysis revealed the dynamics in the *S. coelicolor* M145 metabolite pool composition during growth and stationary/secondary metabolism phases. Preliminary assessment of the global data set by PCA revealed a stronger dependence on cultivation conditions (N lim vs. P lim) since sampling points before the nutrient depletion clustered with respective later samples rather than a time-dependent growth phase clustering (Supplementary Fig. S4).

**Fig. 2:**
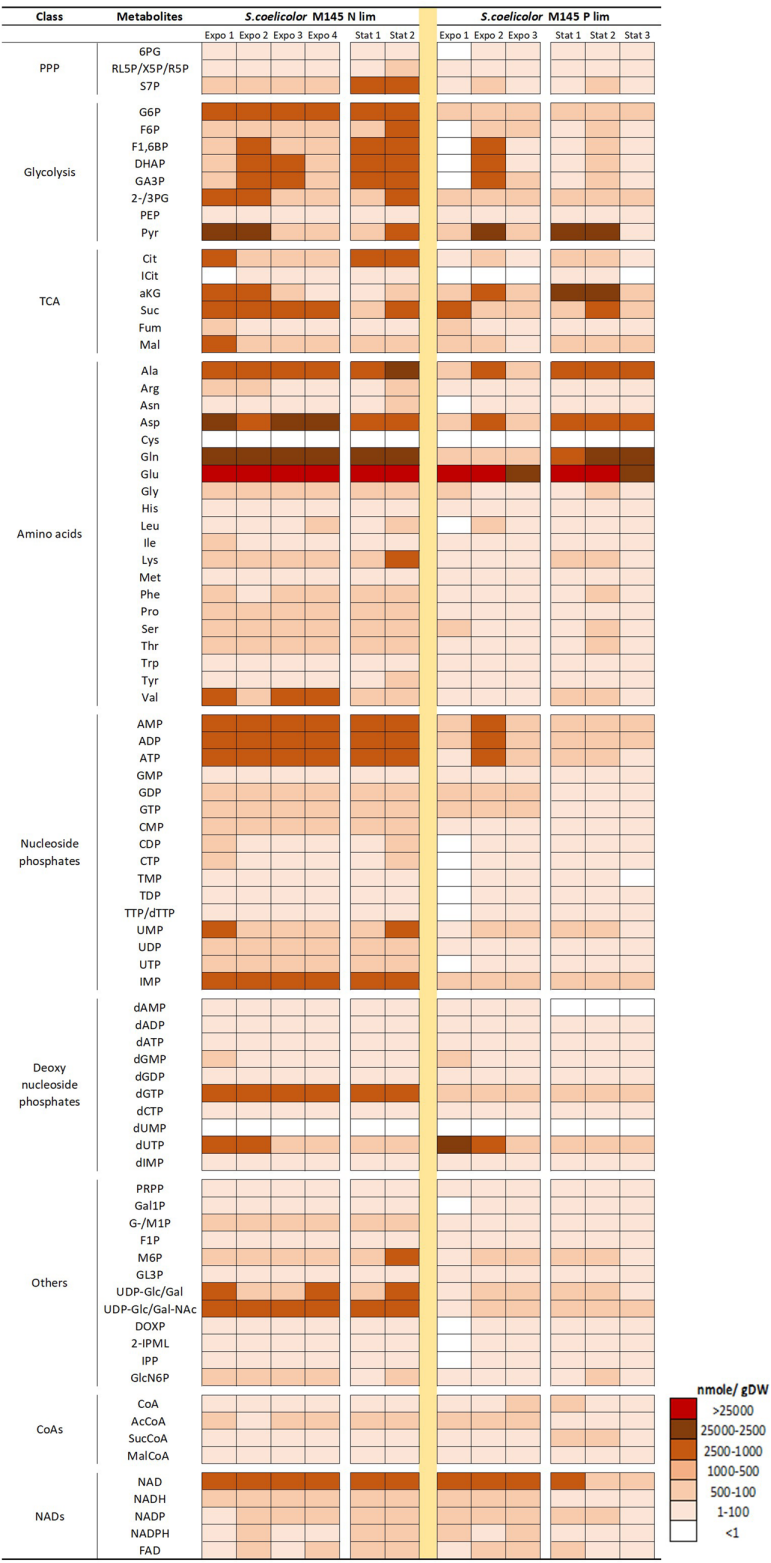
Heat map representation of the absolute concentration of intracellular metabolite pools of *S.coelicolor* M145. White indicates that the metabolites were not analyzed/not included/ trace amount (< 1 nmol/gDCW). R5P is the combination of X5P, R5P, and RL5P. Concentrations and standard deviations can be found in Supplementary Table S2. Supplementary Fig. S6 also shows the heat-map representation of relative standard deviation for two to three samples. Expo and Stat stand for the sample points analyzed from the exponential and the stationary phase, respectively

The intracellular metabolite concentrations varied over 10^4^ orders of magnitude as shown in the time-course heat-map, where metabolites were grouped over seven color concentration intervals (Fig. 2). The relative standard deviation for most of the metabolites was below 30% (Supplementary Table S2 and Fig. S6). Several amino acids (Glutamate—Glu, Glutamine—Gln, Aspartate—Asp, Alanine—Ala) and organic acids (Pyruvate—Pyr and α-ketoglutarate—αKG) were among the highest abundant ones in both cultivation conditions, while there were many low abundant among the glycolytic, deoxynucleoside phosphate, NAD, and CoA metabolites. In general, many metabolite pools decreased in the stationary phase. Of particular interest to note is that the concentration of NAD was much higher than the other NAD metabolites and dropped dramatically in the P lim stationary phase. The culture still maintained the same energy charge levels (ECR). The average ECR for the *S. coelicolor* M145 was 0.48 ± 0.02 (in SSBM-E media), and 0.44 ± 0.07 (in SSBM-P media) (Supplementary Table S1).

**Fig. 3:**
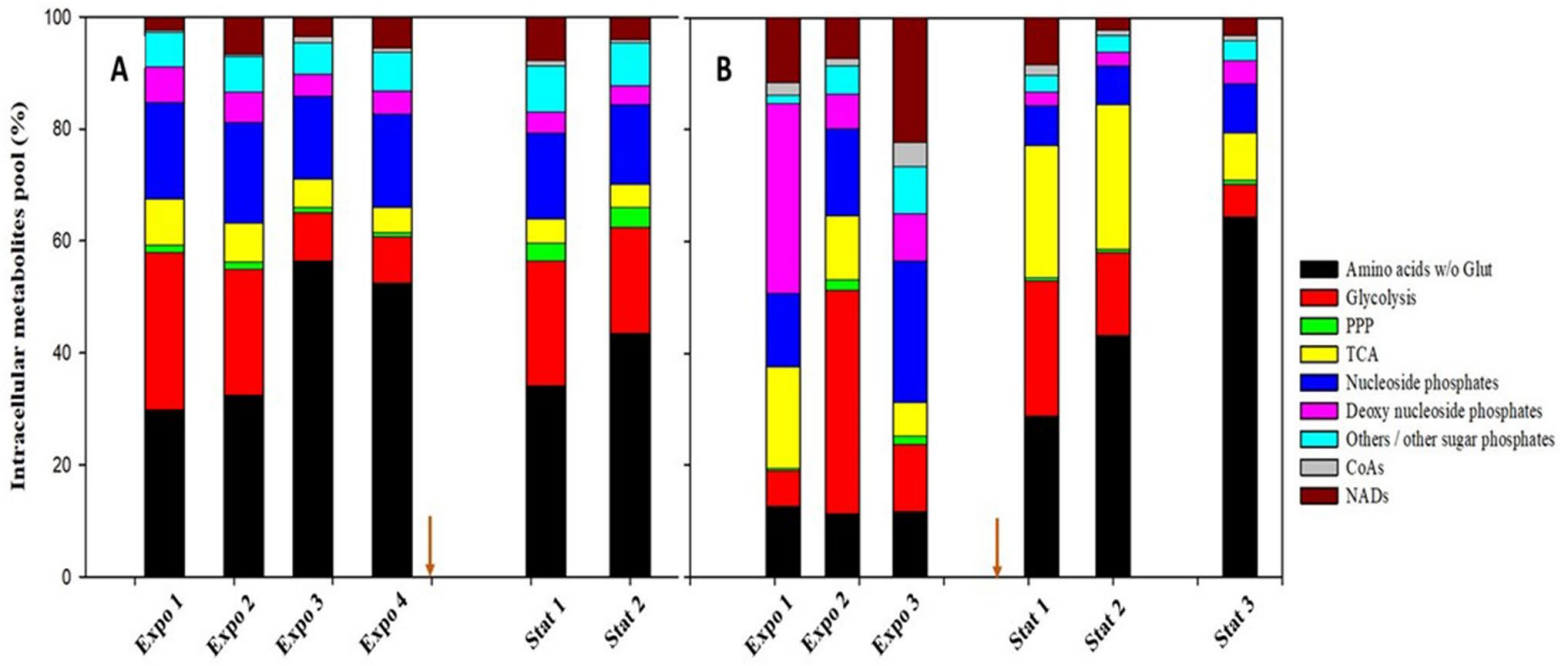
The stacked bar diagram shows the intracellular metabolites pool (excluding glutamate) in the exponential phase and the stationary phase when *S. coelicolor* M145 was cultivated in (**A**) L-glutamate limited and **B** phosphate limited media. The pointed arrow on the X-axis separates the exponential (Expo) and the stationary (Stat) phase

Next, intracellular metabolites were grouped in metabolite classes to draw a better understanding of the effect of cultivation conditions and growth phases. The intracellular Glu level was not included when calculating the percentage composition because of two reasons: first, Glu contributes to most of the concentration of the total metabolites, thus overshadowing the visualization of the pool contribution of the other metabolites, and second, a possible overestimated quantification of Glu due to its contamination from the L-glutamate containing media. Similar to our previous report on *S. coelicolor* M1152 and M1581 strains (Kumar and Bruheim 2021b), a relative downregulation of all the phosphorylated metabolites and upregulation of amino acids were observed after phosphate depletion (Fig. 3). In contrast, L-glutamate depletion did not compromise the phosphorylated metabolite pools, but most noteworthy was the reduction in the TCA cycle metabolite pools succeeding aKG.

The relative change in individual metabolite levels across different time points and cultivation conditions was visualized through another time series heat-map representation. For this purpose, the ratio of the metabolite concentration at individual time points to the average concentration across all sampling points was log2 transformed for each cultivation. In N lim medium, most of the metabolites had a high level at the early exponential phase, decreased around glutamate depletion but recovered at the end of the cultivation (Supplementary Fig. S5). Contrary to N lim, in general, metabolite pools peaked in the mid-exponential phase in P lim medium except for the amino acids that were low in the growth phase but accumulated in the P lim induced stationary phase. Most of the phosphorylated metabolites were decreased after experiencing phosphate depletion. The Fig. S5 plot highlights the dynamics of the central metabolome during growth, transition, and finally secondary metabolism phase.

**Fig. 4:**
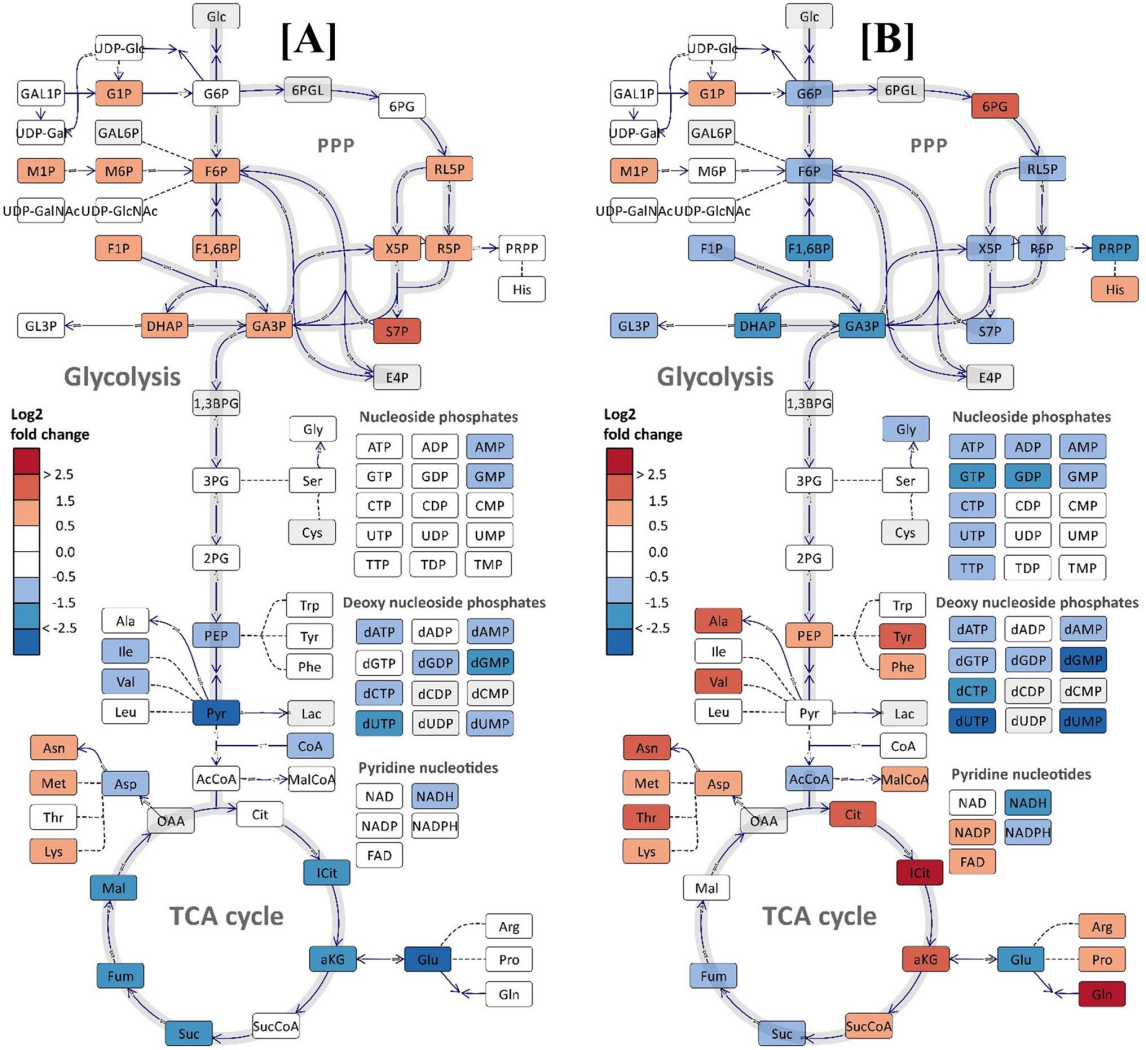
Log2 fold change of central carbon metabolites (CCMs) of *S.coelicolor* M145 corresponding to the maximum levels of metabolites in the stationary phase relative to the exponential phase in (**A**) L-glutamate limited, **B** phosphate limited media. Grey indicates that the metabolites were not analyzed/not included. All metabolites were visualized using the Omix software (Droste et al. 2011)

Inspection and interpretation of such large data sets are challenging, and therefore the highest metabolite levels from each of the exponential and nutrient depletion phases were selected for further assessment of the results. The log2 transformed ratio between the nutrient depletion and exponential phase is presented in the metabolic network perspective in Fig. 4. The overall picture is quite remarkable: upper glycolytic and PPP pools accumulate while lower glycolytic, TCA, and amino acids pools decrease in the N lim cultivation, and the opposite adaption in the metabolite pools are observed in the P lim cultivation (Fig. 4A vs B). It is only the nucleoside phosphate pools that change in the same direction (downregulation) for both cultivations. In N lim of *S. coelicolor* M145, the depletion of L-glutamate in the cultivation broth is not counteracted by upregulation of glucose consumption (Table 1). This led to a downregulation of TCA activity resulting in decreased TCA metabolites and NADH levels, as well as the lower glycolytic metabolites (Fig. 4A). Both carbon sources are available throughout the whole P lim cultivation but a larger decrease in the stationary phase is observed in the glucose consumption rate than the glutamate consumption rate (Table 1). The twice as high (on mole basis) consumption of glutamate might be reflected in the accumulation of TCA and lower glycolytic metabolites.

Industrial *Streptomyces* bioprocesses are mostly designed with phosphate limitations since this supports higher secondary metabolite production rates, as also observed in the current study with defined medium conditions. Therefore, it is interesting to observe that many primary metabolite pools are downregulated in P lim (Fig. 4B) but there is not a single pronounced metabolite that stands out as the sole bottleneck/ limiting agent, neither among carbon precursors, reducing power (NADPH) nor energetic level (i.e. NADH and ATP). The current model system is not a high yield system and the next study should be to apply the same quantitative metabolite profiling methodology on a set of high yield strains with a much larger (e.g. > 10–20%) carbon flux from the substrates to the secondary metabolites. The current study is focused on validating the analytical methodology and verifying that the BGCs deletions of M1146 did not cause negative effects on the physiological level (see final paragraph).

### ^13^C-isotope tracing

A ^13^C-isotope labeling experiment was performed on *S. coelicolor* M145 in the P lim media to investigate how the carbons of D-glucose and L-glutamate were distributed among the CCMs in the growth and the antibiotics production phase. This study could potentially aid in the better understanding of the physiology of *Streptomyces* sp. and in the interpretation of the metabolite profile data described above, as also performed earlier by others, e.g. Obanye and co-workers used radiorespiroometry and reported a correlation between carbon flux through the PPP and production of the antibiotic (Obanye et al. 1996) and Gunnarson and co-workers applied GC–MS-based ^13^C labeled experimentation and discovered a switch from glycolysis to Entner-Doudoroff pathway during the transition from growth to secondary metabolism in the Actinomycete *Nonomuria gerenzanensis* (Gunnarson et al. 2004).

**Fig. 5:**
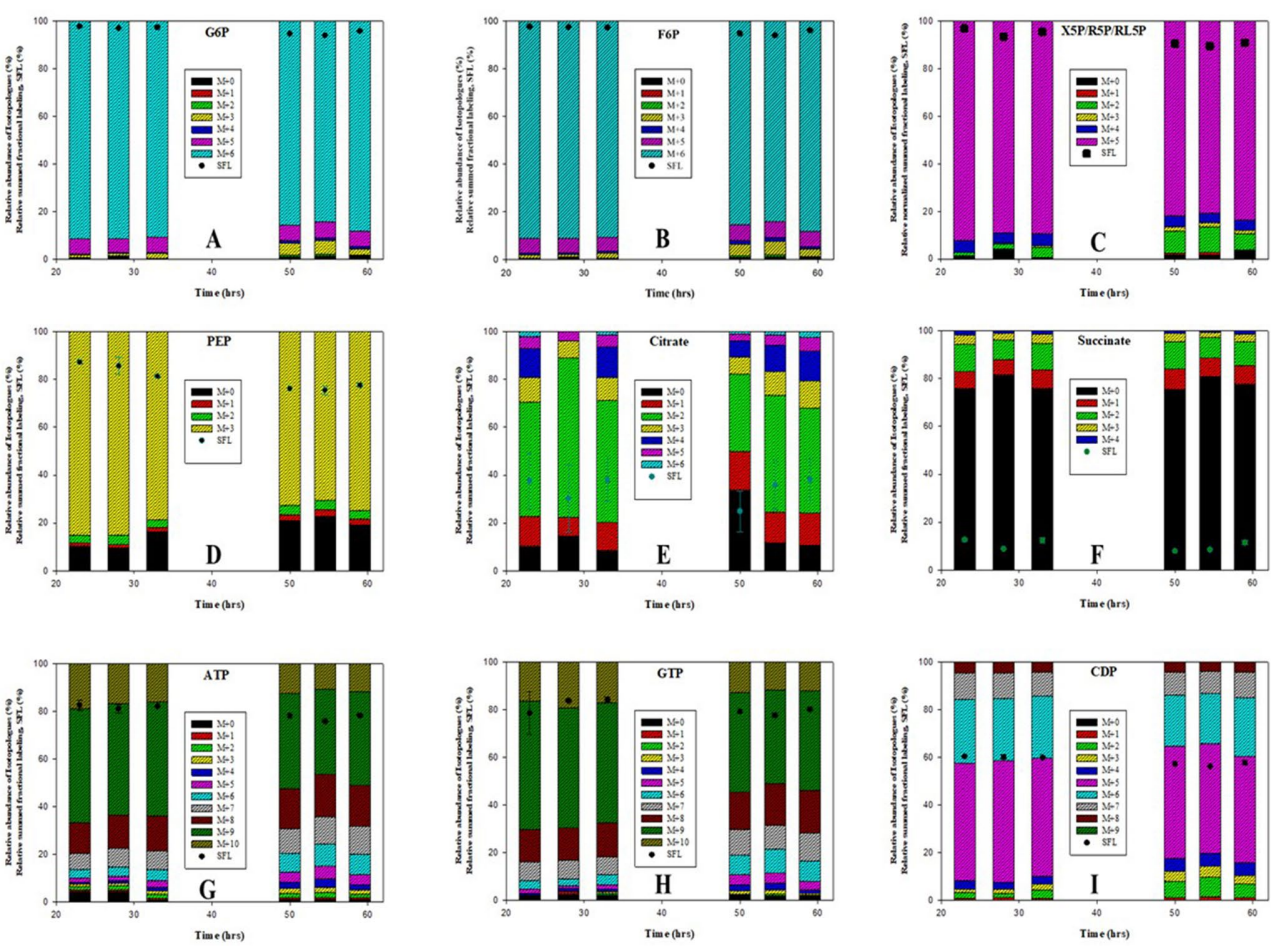
The relative abundance of the isotopologues of the selected metabolites from central carbon metabolites (CCMs) shows the distribution of ^13^C carbon from D-glucose and ^12^C carbon from L-glutamate of *S.coelicolor* M145 in phosphate limited media. **A** Glucose-6-phosphate, G6P; **B** fructose-6-phosphate, F6P; **C** xylulose-5-phosphate/ribose-5-phosphate/ribulose-5-phosphate, X5P/R5P/RL5P; **D** phosphoenolpyruvate, PEP; **E** citrate; **F** succinate; **G** ATP; **H** GTP; **I** CDP. The square dotes in each of the figures show the relative Summed Fractional Labeling (Rel SFL), corresponding to each time point. M + 0, M + 1, M + 2…M + n represent the molecular weight of metabolite isotopologues formed due to incorporation of ^13^C labeled carbons, where n is the maximum number of carbon present in that metabolite

Isotopologues of selected metabolites from central metabolic pathways (upper and lower glycolysis, PPP, TCA cycle, purine, and pyrimidine nucleoside phosphate) showing ^13^C-label enrichment from the U-^13^C_6_-glucose are displayed in Fig. 5. Glucose-6-phosphate, (G6P), Fructose-6-phosphate (F6P), and Xylulose-5-phosphate/Ribose-5-phosphate/Ribulose-5-phosphate (X5P/R5P/RL5P) were almost completely ^13^C-labeled in the growth phase but there was a slight reduction in the stationary phase, which is due to the introduction of ^12^C-carbons from L-glutamate. Interestingly, this was not due to general ^12^C enrichment in all isotopologues but limited to M + 3 for the hexose-phosphate and M + 2 the pentose phosphates which indicates the simultaneous glycolysis and gluconeogenesis (bi-directionality) in these central metabolic pathways where complete ^12^C-C_3_ carbon skeletons (e.g. PEP) from L-glutamate is supplied to glycolysis through anaplerotic reactions connecting the glycolytic pathway and TCA (Coze et al. 2013; Sauer and Eikmanns 2005). Different ^13^C labeling patterns in isotopologues of Citrate (Cit) depended upon the reaction between the labeling pattern of oxaloacetate (OAA) and Acetyl coenzyme A (AcCoA). The cyclic nature of TCA and the simultaneous glycolysis and gluconeogenesis can potentially yield all isotopologues of Cit. However, it could be observed that there was an abundance of isotopologues of M + 0, M + 1, and M + 2. Succinate (Suc) mostly consisted of isotopologue of M + 0 carbon, signifying those metabolites downstream of the α-KG receiving most of the carbons from ^12^C carbon of L-glutamate. Purine (ATP and GTP) had a higher Rel SFL as compared to pyrimidine (CDP) nucleoside phosphates.

In summary, upper glycolytic intermediates and PPP were rich with carbons from D-glucose, and lower glycolytic intermediates and Cit (upstream to α-KG) were rich with carbons from both D-glucose and L-glutamate, whereas intermediates downstream to α-KG were mostly composed of carbons from L-glutamate. The simultaneous bidirectionality of the glycolytic pathway was observed with ^12^C carbons of L-glutamate the following gluconeogenesis whereas ^13^C carbons of D-glucose were directed towards glycolysis. The bidirectionality of the glycolytic pathway also implied that some of the ^12^C carbons reentered into the TCA cycle as revealed by isotopologues of Cit. A progressive enrichment of ^12^C labeled carbon into CCMs coincided with a relative increase of L-glutamate over D-glucose consumption rate with the time of cultivation, especially in the antibiotics production phase.

### Derivative strain M1146 vs reference M145 strain during Plim conditions

The derivative strain M1146 had a similar characteristic CO_2_ evolution profile but grew slightly faster than the parent M145 strain (Table 1 and Supplementary Fig. S7A). As expected, this BGCs deleted strain did not produce the pigments corresponding to the deleted BGCs. A higher specific growth rate of *S. coelicolor* M1146 might be because of a lower and simpler genetic and metabolic burden due to the deletion of four BGCs from this strain (Coze et al. 2013).

Phosphate limitation had nearly a similar effect on metabolite pools on both strains (Supplementary Fig. S7B–E). The metabolite pools were high in the exponential phase and most of the phosphorylated metabolites were sharply downregulated after experiencing the phosphate limitation. Similar to the wild/reference strain in P lim media, αKG and Pyruvate levels were increased in *S. coelicolor* M1146. The phosphate limitation in both the strains resulted in a decrease in the Suc pool in the growth phase and its reverse trend in the production phase. Interestingly, in the phosphate-depleted phase, a higher level of 6PG was observed in M1146 which might be linked to less NADPH consumption in absence of antibiotics production. M1146 had a slightly higher ECR (0.58 ± 0.11) compared to M145 (0.44 ± 0.07) which is in resonance with previous studies where an elevated level of ATP/ADP ratios correlated with improved growth performance (Esnault et al. 2017; Coze et al. 2013).

## Conclusion

High-resolution quantitative metabolite profiles of *Streptomyces coelicolor* have been obtained in the nutrient-limited batch cultivation. The nutrient limitation had a larger effect on the metabolite pool composition compared to the deletion of BGCs. A ^13^C based study revealed the cultivation phase-dependent interplay of D-glucose and L-glutamate carbons into the central carbon metabolism.

## Supplementary Information

Below is the link to the electronic supplementary material.

Supplementary file1 Figure 1: The LC–MS/MS methods covering CCM, Figure S2: NADs optimization graphs, Figure S3: CoAs optimization graphs, Figure S4: PCA plot of S. coelicolor) M145 in N and P lim, Figure S5: Heat maps on intracellular metabolite pools, Figure S6: Heat-map of relative standard deviation (%), Figure S7: Growth and metabolites profiling of S. coelicolor M1146, Table S1: Energy charge ratio, Table S2: metabolite concentrations of M145 and M146 cultivations (PDF 987 KB)

Supplementary file2 (PDF 152 KB)

## Abbreviations

6PGL: 6-Phosphogluconolactone
6PG: Phosphogluconic acid
RL5P: Ribulose-5-phosphate
R5P: Ribose-5-phosphate
X5P: Xylulose-5-phosphate
E4P: Erythrose 4-phosphate
S7P: Sedoheptulose-7-phosphate
G6P: Glucose-6-phosphate
F6P: Fructose-6-phosphate
F1,6BP: Fructose-1,6-bisphosphate
DHAP: Dihydroxyacetone phosphate
G3P: D-Glyceraldehyde 3-phosphate
3PG: 3-Phosphoglyceric acid
2PG: 2-Phosphoglyceric acid
PEP: Phosphoenolpyruvic acid
Pyr: Pyruvic acid
2-HG/OHGlu: 2-Hydroxyglutaric acid
Cit: Citric acid
ICit: Isocitric acid
αKG: α-Ketoglutaric acid
Suc: Succinic acid
Fum: Fumaric acid
Mal: Malic acid
OAA: Oxalacetic acid
Ala: Alanine
Arg: Arginine
Asn: Asparagine
Asp: Aspartic acid
Gln: Glutamine
Glu: Glutamic acid
Gly: Glycine
His: Histidine
Leu: Leucine,
Ile: Isoleucine
Lys: Lysine
Met: Methionine
Phe: Phenylalanine
Pro: Proline
Ser: Serine
Thr: Threonine
Trp: Tryptophane
Tyr: Tyrosine
Val: Valine
AMP: Adenosine monophosphate
ADP: Adenosine diphosphate
ATP: Adenosine triphosphate
GMP: Guanosine monophosphate
GDP: Guanosine diphosphate
GTP: Guanosine triphosphate
CMP: Cytidine monophosphate,
CDP: Cytidine diphosphate
CTP: Cytidine triphosphate
UMP: Uridine monophosphate
UDP: Uridine diphosphate
UTP: Uridine triphosphate
TMP: Thiamine monophosphate
TDP: Thymidine diphosphate
TTP: Thymidine triphosphate
IMP: Inosine monophosphate
ITP: Inosine triphosphate
dAMP: Deoxyadenosine monophosphate
dADP: Deoxyadenosine diphosphate
dATP: Deoxyadenosine triphosphate
dGMP: Deoxyguanosine monophosphate
dGDP: Deoxyguanosine diphosphate
dGTP: Deoxyguanosine triphosphate
dCTP: Deoxycytosine triphosphate
dUMP: Deoxyuridine monophosphate
dUTP: Deoxyuridine triphosphate
dTTP: Deoxythymidine triphosphate
dIMP: Deoxyinosine monophosphate
cAMP: Cyclic monophosphate
PRPP: Phosphoribosyl-pyrophosphate
GAL1P: Galactose-1-phosphate
GAL6P: Galactose-6-phosphate
G1P: Glucose-1-phosphate, G1P
M1P: Mannose-1-phosphate
F1P: Fructose-1-phosphate
M6P: Mannose-6-phosphate
GL3P: Glycerol-3-phosphate
UDP-glu: UDP-glucose
UDP-Glc-Nac: UDP-acetylglucosamine
DOXP: Deoxyxylulose 5-Phosphate
2-IPML: 2-Isopropylmalic acid
IPP: Isopentenyl pyrophosphate
GA6P: Glucosamine 6-phosphate
GlcN6P: N-Acetyl-glucosamine 6-phosphate
Lac: L-Lactic acid
T6P: Trehalose 6-phosphate
CoA: Coenzyme A
AcCoA: Acetyl coenzyme A
Mal CoA: Malonyl coenzyme A
Suc CoA: Succinyl coenzyme A
NAD: Nicotinamide adenine dinucleotide (oxidised)
NADH: Nicotinamide adenine dinucleotide (reduced)
NADP: Nicotinamide adenine dinucleotide phosphate (oxidised)
NADPH: Nicotinamide adenine dinucleotide phosphate (reduced)
FAD: Flavin adenine dinucleotide

## Declarations

### Research involved with human or animals participants

The research does not involve human participants or animals. Both authors consent submission of this study.
