# Supplementary material for: Nutrient-depended metabolic switching during batch cultivation of Streptomyces coelicolor explored with absolute quantitative mass spectrometry-based metabolite profiling
Source: 3 Biotech. 2022 Feb 26;12(3):80. doi: 10.1007/s13205-022-03146-x (PMC8882213; doi:10.1007/s13205-022-03146-x)
Supplement: Supplementary file 1 — Supplementary file1 Figure 1: The LC–MS/MS methods covering CCM, Figure S2: NADs optimization graphs, Figure S3: CoAs optimization graphs, Figure S4: PCA plot of S. coelicolor) M145 in N and P lim, Figure S5: Heat maps on intracellular metabolite pools, Figure S6: Heat-map of relative standard deviation (%), Figure S7: Growth and metabolites profiling of S. coelicolor M1146, Table S1: Energy charge ratio, Table S2: metabolite concentrations of M145 and M146 cultivations (PDF 987 KB) [file 13205_2022_3146_MOESM1_ESM.pdf]

Supplementary materials

Nutrient depended metabolic switching of *Streptomyces coelicolor* explored with absolute quantitative mass spectrometry based metabolite profiling

Figure S1. Different colors are used to show the LC-MS/MS methods used for the absolute quantification of central carbon metabolites (CCMs).

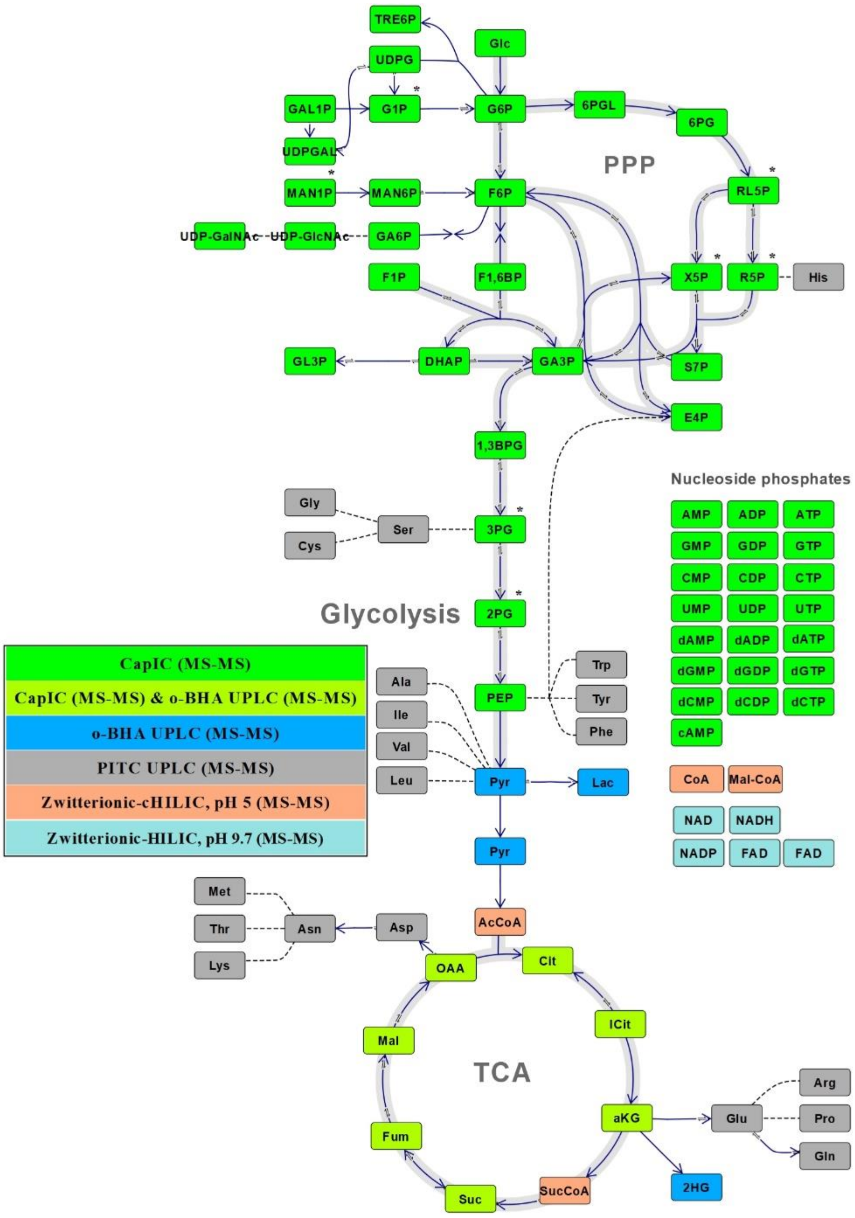

Figure S2. NAD metabolites optimization using biomass of *S.coelicolor* strain **A)** The stability of NAD metabolites overtime when samples were kept in an autosampler at 6 ^{o}C. The change in signal intensity is shown in percentage compared to the area of the 0 hr sample. **B)** The left and right-side figures show signal intensity over time percentage compared to area and response, respectively. The sample was diluted with 10% ISTD. **C).** The figure shows signals of different NAD metabolites when different extraction methods were applied to the same pellet (6.9 mg dry biomass). **D)** Graph shows the linearity of NAD metabolites signals over different pellets sizes. NAD metabolites were extracted from the sample using the hot extraction method (4 minutes at 80 ^{o}C). The signals of NADH and NADPH were not visible in pellets of small sample sizes. **E)** The same biomass pellet (6.9 mg dry biomass) was extracted three times, following the cold extraction (4 ^{o}C) method. The change in signal intensity is shown in percentage compared to the sum of the area of respective metabolites over three extractions.

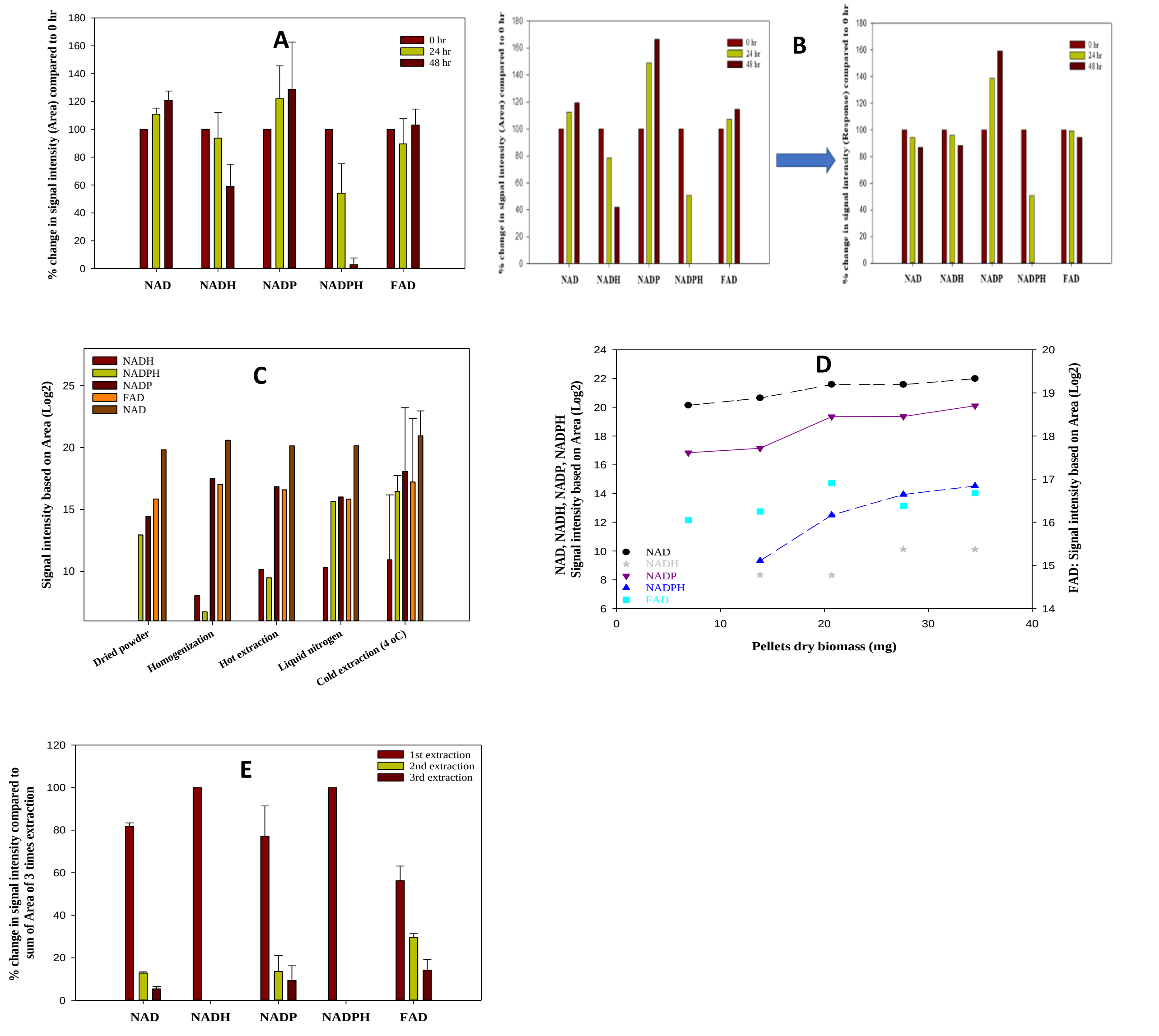

Figure S3. CoA metabolites optimization using biomass of *S.coelicolor* strain **A)** The effect of cold extraction time **B)** sonication time **C)** different extraction methods. **D)** The same pellet (6.9 mg dry biomass) was extracted five times using the cold extraction (4 ^{o}C) method. **E)** The percentage recovery of CoA metabolites after pooling three consecutive extracts. **F)** Effect of two different matrices (prepared by pooling CoA metabolites extract) on the slope of standard curve of each CoA metabolite.

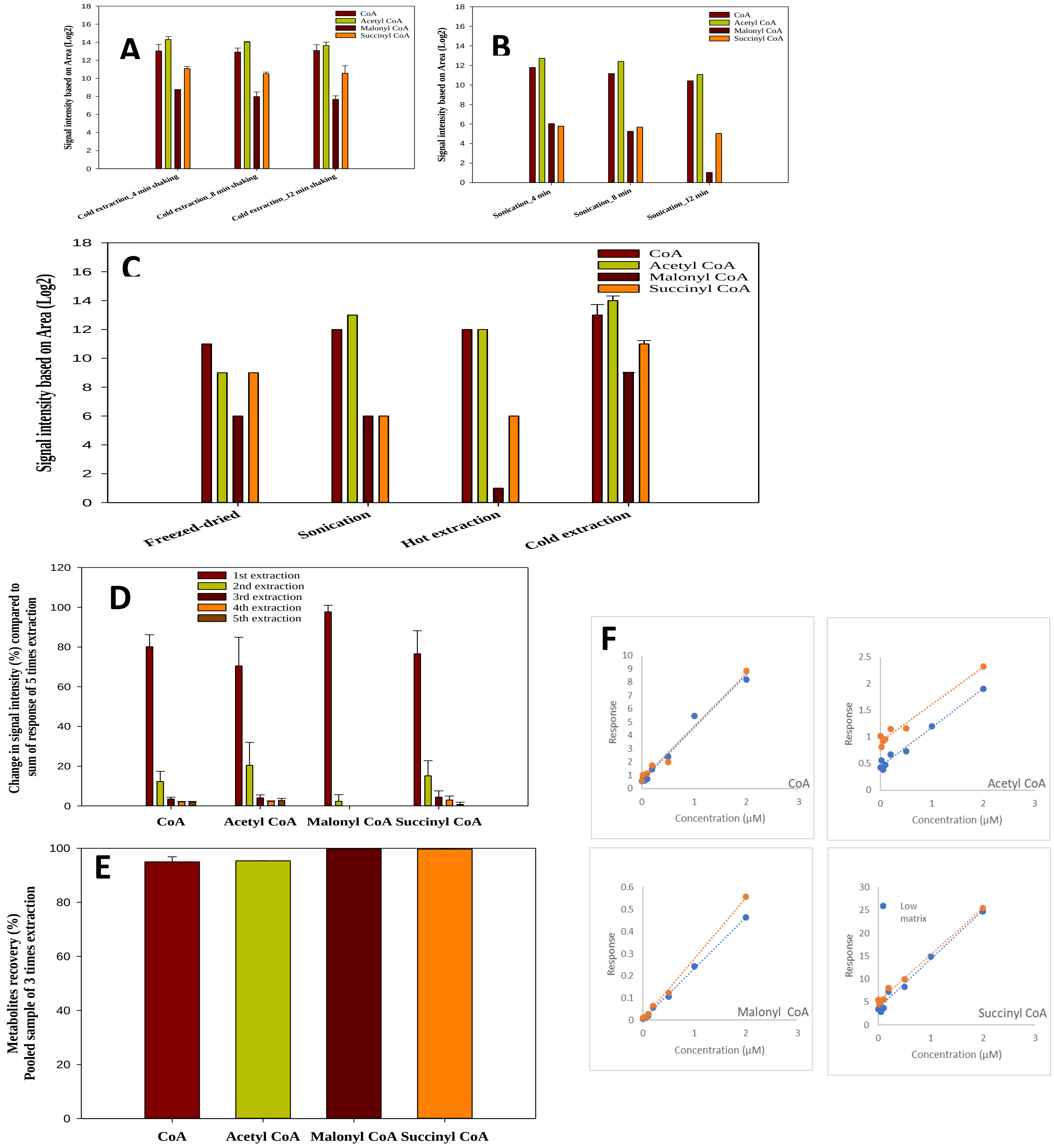

Figure S4: Scores plot from Principal component analysis (PCA) of *Streptomyces coelicolor* M145 N lim and P lim metabolite time series data. Missing values were replaced by feature mean and autoscaled before PCA. MetaboAnalyst software was used for this task

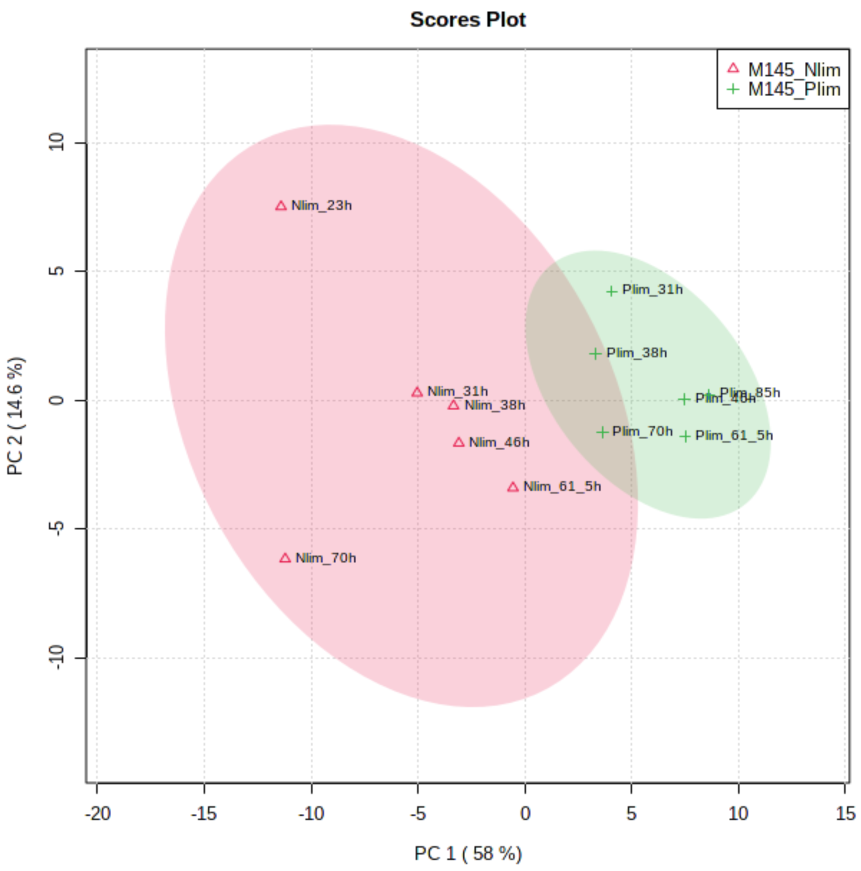

Figure S5. Heat map representation of intracellular metabolite pools of *S.coelicolor* M145. The ratio of the metabolite concentration to the average concentration across all sample points for each cultivation has been log2 transformed to visualize the relative change for the cultivation. Red indicates a high concentration and green indicates a low relative metabolite concentration. Grey indicates that the metabolites were not analyzed/not included/ trace amount. Expo and Stat stand for the sample points analyzed from the exponential and the stationary phase, respectively.

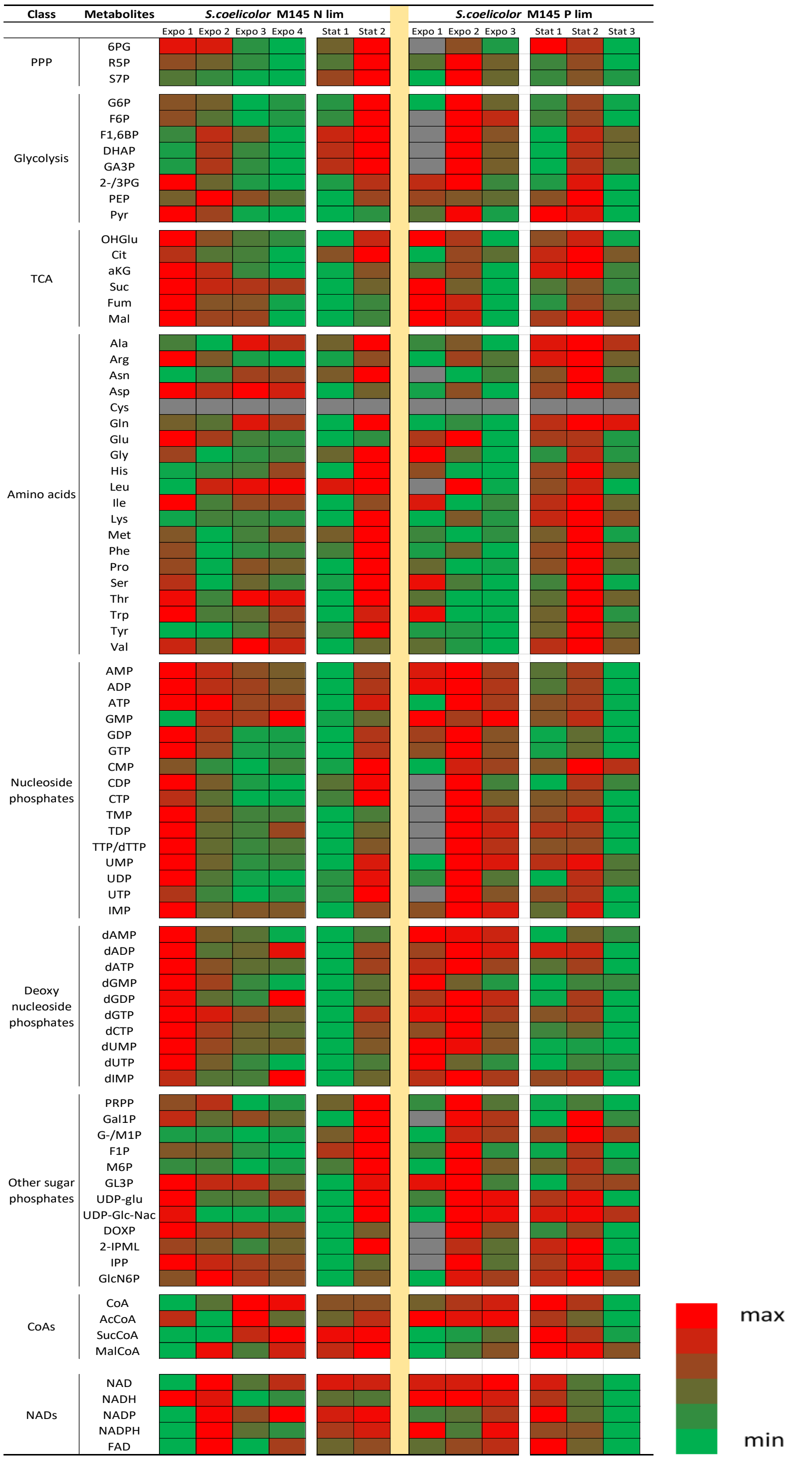

Figure S6. Heat-map showing relative standard deviation (%) for two to three samples.

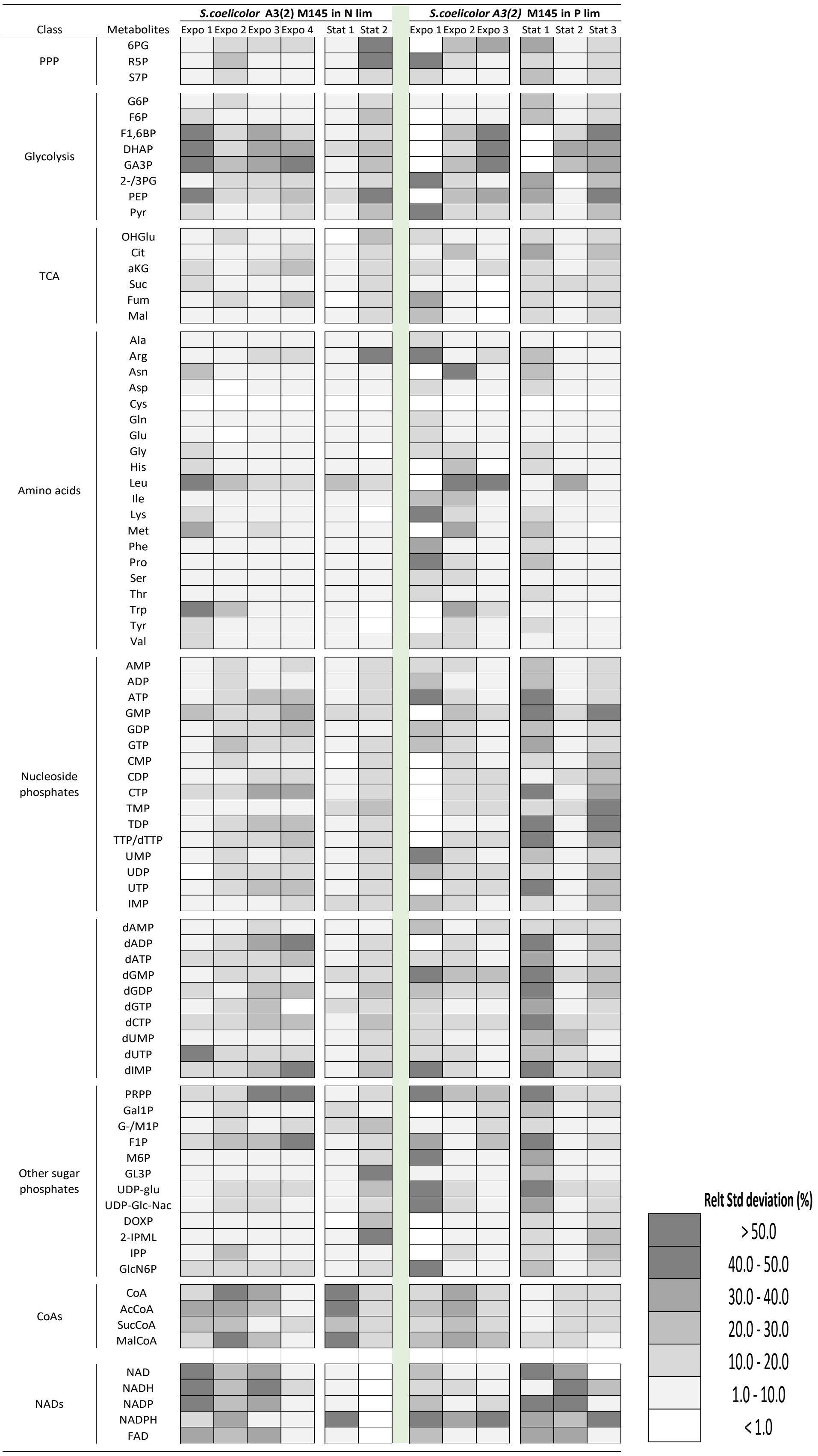

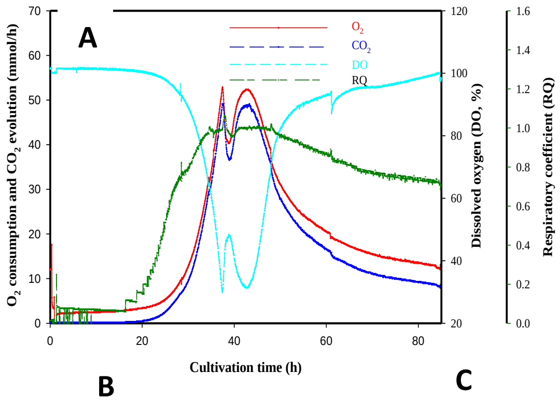

Figure S7. Growth and intracellular metabolites levels of *S. coelicolor* M1146 in phosphate limited media. Expo and Stat stand for the sample points analyzed from the exponential and the stationary phase, respectively. Grey indicates that the metabolites were not analyzed/not included. **A)** Cultivation parameters. **B)** Heat map representation of the absolute concentration of intracellular metabolite pools. White indicates that the metabolites were not analyzed/not included/ trace amount. **C)** Heat map representation of the ratio of the metabolite concertation to the average concentration across all sample points has been log2 transformed to visualize the relative change for the cultivation. Red indicates a high concentration and green indicates a low relative metabolite concentration. **D)** The stacked bar diagram of the intracellular metabolites pool (excluding glutamate) at each sampling point. The pointed arrow on the X-axis separates the exponential and the stationary growth phase. **E)** Log2 fold change of central carbon metabolites corresponding to the maximum level of metabolites in the stationary phase relative to the exponential phase. All metabolites were visualized using the Omix software (Droste et al., 2011).

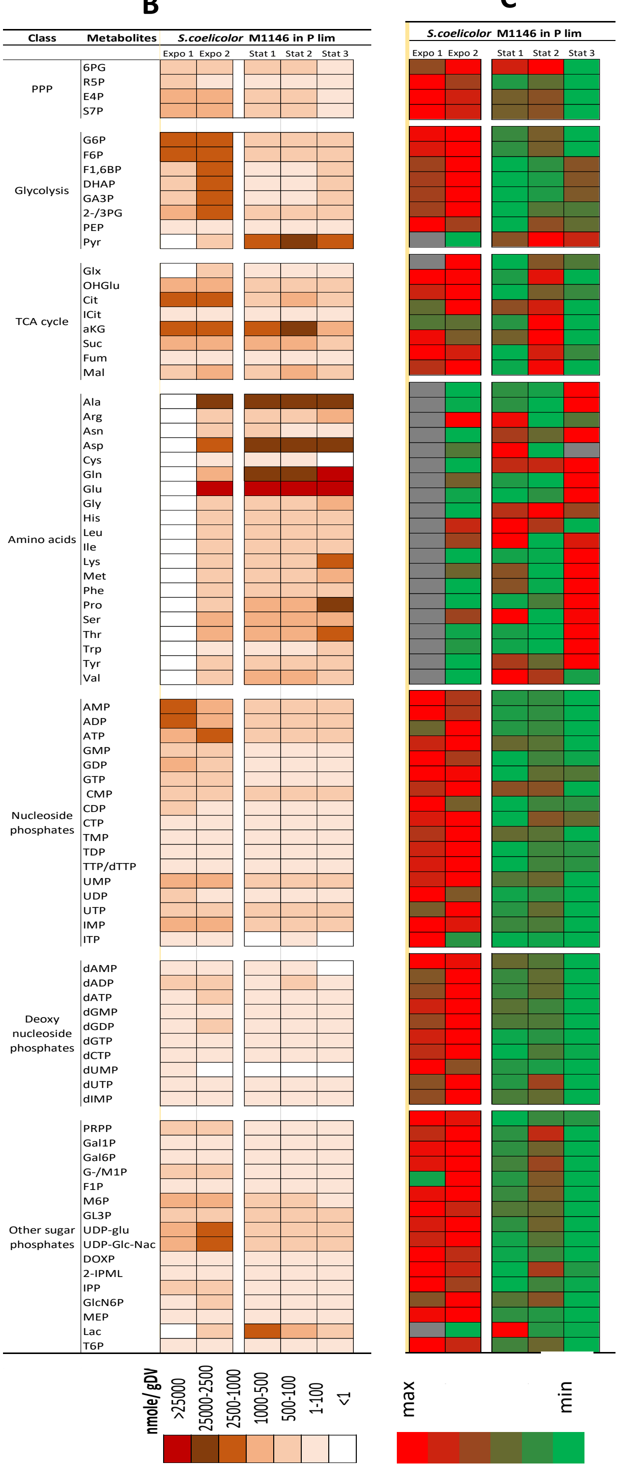

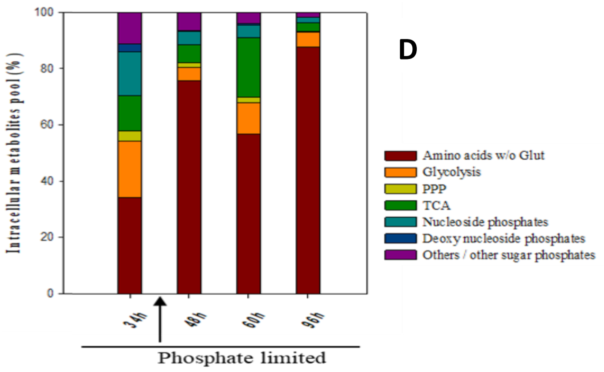

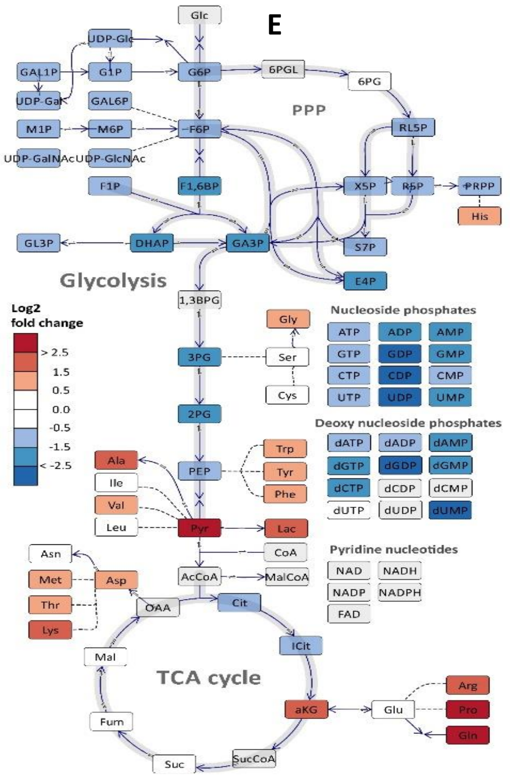

Table S1. The average energy charge ratio (ECR) calculated across the cultivation time points in the *S. coelicolor* M145 in L-Glutamate and phosphate limited media, and in the *S. coelicolor* M1146 in the phosphate limited media.

***S. coelicolor* M145** (SSBM-E)		***S. coelicolor* M145** (SSBM-P)		***S. coelicolor* M1146** (SSBM-P)	
Time (h)	ECR	Time (h)	ECR	Time (h)	ECR
23	0.44	31	0.32	28	0.38
31	0.48	38	0.49	34	0.64
38	0.47	46	0.46	48	0.62
46	0.49	61.5	0.49	60	0.62
61.5	0.49	70	0.46	96	0.64
70	0.48	85	0.41		
Average	0.48 ± 0.02		0.44 ± 0.07		0.58 ± 0.11
