# Supplementary material for: Nutrient-depended metabolic switching during batch cultivation of Streptomyces coelicolor explored with absolute quantitative mass spectrometry-based metabolite profiling
Source: 3 Biotech. 2022 Feb 26;12(3):80. doi: 10.1007/s13205-022-03146-x (PMC8882213; doi:10.1007/s13205-022-03146-x)
Supplement: Supplementary file 2 — Supplementary file2 (PDF 152 KB) [file 13205_2022_3146_MOESM2_ESM.pdf]

Supplementary Table S2 Manuscript: Nutrient depended metabolic switching of *Streptomyces coelicolor* explored with absolute quantitative mass spectrometry based metabolite profiling

Metabolite concentrations

Units: nmole per mg DCW

		M145_Nlim												M145_Plim												M1146_Plim									
		23h		31h		38h		46h		61_5h		70h		31h		38h		46h		61_5h		70h		85h		28		34		48		60		96	
		Avg	Rel Stdv (%)	Avg	Rel Stdv (%)	Avg	Rel Stdv (%)	Avg	Rel Stdv (%)	Avg	Rel Stdv (%)	Avg	Rel Stdv (%)	Avg	Rel Stdv (%)	Avg	Rel Stdv (%)	Avg	Rel Stdv (%)	Avg	Rel Stdv (%)	Avg	Rel Stdv (%)	Avg	Rel Stdv (%)	Avg	Rel Stdv (%)	Avg	Rel Stdv (%)	Avg	Rel Stdv (%)	Avg	Rel Stdv (%)	Avg	Rel Stdv (%)
PPP	6PG	0.079	9.8	0.073	12.9	0.022	18.0	0.016	11.9	0.036	1.5	0.095	40.9			0.009	26.0	0.003	34.4	0.025	37.2	0.012	5.3	0.002	14.4	0.100	2.9	0.366	9.6	0.220	2.5	0.390	0.6	0.016	5.4
	R5P	0.094	6.4	0.080	21.9	0.056	7.4	0.043	6.5	0.068	6.1	0.171	41.1	0.017	75.3	0.071	16.2	0.021	9.6	0.014	18.7	0.028	6.8	0.008	16.3	0.154	3.1	0.063	0.7	0.017	2.5	0.032	1.4	0.012	0.8
	S7P	0.399	1.6	0.321	10.4	0.225	7.6	0.214	7.2	0.675	2.8	1.431	15.0	0.041	14.8	0.264	4.6	0.090	4.4	0.063	25.2	0.108	1.8	0.053	13.0	0.995	4.3	0.603	1.5	0.217	1.4	0.270	1.7	0.059	1.6
Glycolysis	G6P	0.866	3.5	0.822	10.3	0.593	7.4	0.658	7.8	0.663	1.9	1.207	13.6	0.140	8.6	0.431	2.1	0.227	3.9	0.178	22.7	0.279	2.0	0.149	11.8	1.292	6.2	1.479	0.8	0.202	1.2	0.380	3.1	0.114	2.3
	F6P	0.396	19.1	0.325	8.9	0.236	6.3	0.268	8.1	0.285	4.7	0.596	26.9			0.162	6.4	0.126	8.3	0.076	23.1	0.107	8.2	0.058	11.7	1.237	8.8	1.718	6.4	0.206	4.7	0.421	3.5	0.127	8.9
	F1,6BP	0.241	75.6	0.876	13.2	0.416	38.3	0.139	19.2	0.965	7.1	1.624	17.5			0.695	28.7	0.058	48.6	0.003		0.197	18.7	0.034	76.4	0.355	4.2	1.085	1.0	0.054	3.2	0.093	1.4	0.267	3.5
	DHAP	0.400	41.2	1.130	19.5	0.502	30.5	0.334	37.0	1.256	10.2	2.019	22.1			1.006	17.5	0.091	43.5	0.011		0.293	31.3	0.068	30.3	0.408	4.1	1.120	7.3	0.063	2.7	0.086	4.5	0.280	0.4
	GA3P	0.456	41.3	1.244	22.8	0.520	32.0	0.375	41.2	1.329	9.1	2.122	23.1			1.080	23.2	0.107	41.9	0.014		0.313	30.0	0.067	35.3	0.421	5.0	1.177	8.5	0.067	4.5	0.095	3.8	0.278	1.8
	2-/3PG	0.941	3.1	0.511	11.9	0.374	13.2	0.328	13.1	0.371	2.4	0.700	14.4	0.289	77.1	0.407	13.4	0.141	5.9	0.123	30.6	0.345	1.0	0.103	21.6	0.734	1.4	1.852	2.8	0.148	3.9	0.340	1.8	0.322	4.9
	PEP	0.020	75.9	0.052	12.5	0.023	16.2	0.016	20.4	0.009	18.1	0.026	49.8	0.016		0.013	26.6	0.010	30.8	0.014	35.8	0.034	2.3	0.005	77.9	0.042	4.1	0.021	0.7	0.006	3.0	0.018	4.4	0.013	3.8
	Pyr	10.386	14.0	2.816	6.3	0.398	4.5	0.334	14.2	0.337	8.0	0.599	21.1	0.350	63.5	3.786	11.2	0.141	10.2	4.082	17.2	2.540	9.2	0.098	25.5			0.233	NA	1.095	NA	4.147	NA	2.243	NA
TCA	OHGlu	1.015	4.8	0.525	10.8	0.370	5.3	0.317	8.4	0.235	0.7	0.737	29.2	0.770	16.4	0.382	4.3	0.086	2.7	0.295	19.1	0.500	3.4	0.096	16.8	0.555	4.2	0.550	1.6	0.155	1.4	0.476	0.6	0.131	1.9
	Cit	0.629	5.4	0.410	9.5	0.385	4.3	0.283	11.1	0.513	3.6	0.860	13.2	0.021	3.5	0.115	22.1	0.061	7.8	0.194	30.4	0.368	4.8	0.087	21.0	1.090	6.9	1.518	2.1	0.282	2.4	0.622	3.1	0.411	4.1
	aKG	1.062	10.3	0.548	5.8	0.146	13.5	0.082	23.4	0.087	5.9	0.320	11.1	0.415	18.0	1.267	7.6	0.108	18.7	3.637	13.3	5.939	7.4	0.296	14.4	1.766	5.4	1.949	2.1	1.202	3.8	7.815	3.2	0.856	1.3
	Suc	1.467	12.7	0.967	7.7	0.835	7.5	0.790	5.4	0.228	7.7	0.518	16.1	1.371	8.6	0.485	7.9	0.201	0.6	0.367	17.6	0.563	15.0	0.302	10.8	0.891	3.3	0.574	6.3	0.560	3.5	0.939	3.3	0.362	0.7
	Fum	0.181	3.1	0.079	12.9	0.080	7.1	0.036	25.3	0.032	0.7	0.048	16.8	0.115	38.5	0.077	8.8	0.015	0.8	0.021	16.0	0.052	5.3	0.031	17.8	0.088	3.7	0.066	6.1	0.016	0.5	0.066	3.3	0.023	3.9
	Mal	0.655	8.1	0.341	9.5	0.340	4.4	0.119	5.5	0.120	3.5	0.180	16.5	0.318	26.9	0.233	3.1	0.060	0.9	0.180	18.1	0.302	4.5	0.129	16.2	0.350	5.8	0.563	2.9	0.108	2.1	0.527	2.0	0.101	3.6
Amino acids	Ala	1.350	8.0	1.048	6.0	2.358	6.1	2.002	1.2	1.567	2.2	2.580	5.5	0.295	19.9	0.560	9.1	0.174	6.4	1.361	9.2	1.821	0.9	0.930	5.2			6.996	NA	8.040	NA	7.489	NA	15.092	NA
	Arg	0.138	3.5	0.102	3.8	0.081	16.5	0.076	17.3	0.080	5.3	0.107	83.3	0.006	173.2	0.028	6.7	0.014	11.5	0.050	24.4	0.067	4.0	0.019	3.5			0.165	NA	0.203	NA	0.159	NA	0.642	NA
	Asn	0.036	26.6	0.049	6.0	0.090	6.3	0.085	3.0	0.072	3.7	0.152	2.8	0.000		0.004	43.3	0.007	9.1	0.010	24.2	0.021	6.3	0.007	9.1			0.145	NA	0.140	NA	0.074	NA	0.093	NA
	Asp	3.736	4.0	2.355	0.9	3.715	8.0	2.769	5.0	0.685	4.1	1.472	1.3	0.295	15.1	0.765	5.5	0.249	5.8	0.873	10.8	1.875	2.9	0.817	4.4			1.925	NA	3.792	NA	2.934	NA	5.371	NA
	Cys																											0.029	NA	0.035	NA	0.026	NA	0.000	NA
	Gln	6.643	2.7	5.851	1.3	11.069	5.1	8.529	2.3	3.811	2.8	12.845	4.7	0.115	16.7	0.247	6.6	0.110	5.2	2.267	4.6	6.636	1.8	4.067	4.5			0.890	NA	11.832	NA	12.769	NA	27.064	NA
	Glu	975.063	1.4	306.241	0.7	83.313	6.4	63.252	3.7	34.445	2.0	63.850	5.5	200.511	18.7	674.564	2.9	14.038	4.5	176.761	9.1	204.504	1.9	22.839	9.8			102.110	NA	92.591	NA	89.346	NA	119.276	NA
	Gly	0.312	13.4	0.181	1.4	0.212	4.9	0.227	4.4	0.262	2.6	0.435	0.5	0.263	15.2	0.085	12.2	0.044	4.7	0.060	10.0	0.171	3.5	0.058	2.2			0.395	NA	0.382	NA	0.422	NA	0.675	NA
	His	0.041	20.0	0.044	3.3	0.045	8.6	0.052	5.2	0.040	4.3	0.062	3.3	0.019	0.0	0.008	24.7	0.007	0.0	0.021	12.5	0.041	1.8	0.015	4.3			0.113	NA	0.154	NA	0.173	NA	0.146	NA
	Leu	0.004	141.4	0.066	20.9	0.099	13.5	0.123	12.7	0.082	22.8	0.132	14.9			0.114	146.6	0.024	41.6	0.057	2.6	0.083	39.9	0.023	7.5			0.299	NA	0.400	NA	0.267	NA	0.109	NA
	Ile	0.127	3.8	0.055	4.5	0.078	8.7	0.079	5.5	0.040	7.6	0.077	4.1	0.045	24.7	0.008	24.7	0.012	4.9	0.035	9.4	0.060	3.8	0.018	6.3			0.322	NA	0.371	NA	0.256	NA	0.352	NA
	Lys	0.271	12.7	0.336	6.0	0.324	4.5	0.302	3.0	0.254	3.1	0.708	0.4	0.029	47.1	0.077	10.6	0.039	8.2	0.134	10.4	0.207	5.3	0.084	2.0			0.359	NA	0.410	NA	0.376	NA	1.973	NA
	Met	0.052	36.5	0.030	8.3	0.041	12.5	0.052	7.8	0.050	2.6	0.089	7.4	0.019	0.0	0.014	35.3	0.018	8.8	0.026	25.7	0.051	5.2	0.016	0.0			0.352	NA	0.390	NA	0.231	NA	0.613	NA
	Phe	0.146	3.3	0.098	5.1	0.112	9.8	0.115	4.2	0.118	5.1	0.198	1.0	0.032	34.6	0.057	8.6	0.027	3.8	0.073	15.5	0.148	4.0	0.058	1.1			0.318	NA	0.392	NA	0.322	NA	0.470	NA
	Pro	0.152	3.1	0.103	2.4	0.147	9.2	0.139	6.3	0.102	1.8	0.200	2.1	0.026	43.3	0.014	13.3	0.015	6.7	0.032	20.4	0.061	6.5	0.024	2.7			0.499	NA	0.554	NA	0.966	NA	5.073	NA
	Ser	0.395	4.4	0.258	2.0	0.331	6.4	0.297	5.5	0.271	4.2	0.465	4.5	0.244	19.9	0.098	11.6	0.064	2.8	0.093	5.2	0.271	1.7	0.067	4.2			0.679	NA	0.740	NA	0.590	NA	0.736	NA
	Thr	0.450	8.3	0.286	2.5	0.457	4.3	0.440	2.7	0.244	3.4	0.466	5.1	0.070	15.7	0.039	8.3	0.039	2.6	0.073	13.8	0.211	3.1	0.082	1.4			0.699	NA	0.709	NA	0.681	NA	1.053	NA
	Trp	0.030	41.7	0.018	20.8	0.019	6.3	0.024	4.6	0.015	6.7	0.027	0.0	0.019	0.0	0.005	34.6	0.006	10.8	0.010	8.6	0.021	3.5	0.007	0.0			0.064	NA	0.061	NA	0.058	NA	0.124	NA
	Tyr	0.064	15.1	0.064	2.3	0.078	7.2	0.095	3.6	0.074	1.3	0.130	0.0	0.019	0.0	0.019	16.7	0.016	3.8	0.031	11.4	0.076	3.4	0.028	2.3			0.107	NA	0.162	NA	0.138	NA	0.199	NA
	Val	0.533	12.1	0.350	1.8	0.671	4.6	0.531	5.2	0.220	1.8	0.347	3.9	0.070	15.7	0.041	19.9	0.031	7.0	0.153	6.4	0.277	5.5	0.091	9.9			0.449	NA	0.751	NA	0.637	NA	0.471	NA
Nucleoside phosphates	AMP	1.621	5.4	1.277	10.3	1.040	3.7	0.959	12.2	0.599	6.2	1.143	12.0	0.470	18.0	0.551	12.0	0.371	4.8	0.267	28.2	0.376	1.9	0.181	13.4	1.597	6.6	0.776	6.4	0.222	2.5	0.253	6.0	0.162	1.3
	ADP	1.638	1.4	1.229	11.8	1.103	5.7	0.957	6.9	0.561	1.3	1.173	15.2	0.485	26.8	0.521	8.9	0.379	4.8	0.259	21.8	0.359	7.8	0.186	19.7	1.564	8.5	0.772	5.3	0.205	0.8	0.243	1.5	0.159	2.3
	ATP	1.117	5.3	1.122	15.8	0.859	22.7	0.875	24.2	0.568	2.7	1.008	11.4	0.097	58.1	0.529	10.1	0.295	6.9	0.245	40.4	0.298	4.2	0.099	19.9	0.668	3.3	1.637	7.3	0.430	5.8	0.483	1.2	0.345	5.5
	GMP	0.030	23.1	0.056	15.2	0.053	12.7	0.072	31.7	0.031	12.1	0.043	11.9	0.042		0.025	21.2	0.042	13.2	0.021	59.0	0.029	13.9	0.010	43.3	0.211	7.7	0.358	4.7	0.081	6.8	0.069	11.7	0.029	11.0
	GDP	0.413	5.2	0.306	16.2	0.182	12.6	0.183	24.0	0.168	2.6	0.314	10.0	0.125	22.2	0.240	13.1	0.102	1.1	0.042	26.6	0.078	4.1	0.039	17.3	0.515	5.1	0.204	2.7	0.032	3.3	0.063	5.6	0.037	1.9
	GTP	0.389	5.8	0.278	24.0	0.179	13.1	0.182	18.8	0.164	2.3	0.302	12.1	0.105	23.8	0.238	11.1	0.101	2.6	0.043	30.9	0.076	4.7	0.037	17.4	0.378	3.5	0.348	9.5	0.027	3.3	0.074	3.1	0.065	6.4
	CMP	0.164	3.7	0.137	10.7	0.123	4.8	0.141	7.0	0.129	0.7	0.215	15.6	0.017		0.050	11.1	0.039	5.9	0.033	14.0	0.063	8.0	0.044	20.1	0.271	5.3	0.379	0.9	0.216	2.1	0.213	5.3	0.109	2.2
	CDP	0.161	5.2	0.086	9.5	0.056	13.8	0.048	19.7	0.074	3.3	0.155	15.3	0.000		0.044	15.4	0.020	17.7	0.015	6.1	0.032	16.1	0.019	22.4	0.168	3.2	0.026	3.6	0.005	1.7	0.012	1.8	0.010	4.0
	CTP	0.122	10.1	0.089	16.8	0.066	30.6	0.068	39.2	0.083	3.4	0.153	14.7			0.039	18.7	0.017	17.4	0.018	73.0	0.020	4.4	0.008	32.0	0.078	3.7	0.100	3.1	0.018	3.9	0.044	0.9	0.036	3.7
	TMP	0.013	6.5	0.007	6.2	0.006	5.4	0.006	5.5	0.004	15.1	0.006	28.5			0.004	12.9	0.002	11.7	0.002	14.1	0.003	19.6	0.001	42.3	0.026	5.9	0.061	4.8	0.011	1.8	0.010	7.0	0.004	0.7
	TDP	0.056	5.0	0.030	17.0	0.026	23.5	0.037	28.4	0.020	4.0	0.031	14.5			0.017	6.5	0.011	5.5	0.009	60.6	0.008	7.6	0.002	66.7	0.046	3.9	0.074	3.3	0.006	0.7	0.011	1.7	0.009	1.5
	TTP/dTTP	0.052	5.4	0.027	18.1	0.023	18.0	0.025	28.4	0.018	5.0	0.031	13.7			0.019	17.6	0.009	10.6	0.005	46.7	0.006	1.3	0.001	33.0	0.041	3.7	0.059	0.7	0.005	3.1	0.010	2.4	0.008	1.6
	UMP	0.740	5.6	0.490	12.8	0.406	9.5	0.424	10.8	0.357	2.9	0.665	17.2	0.036	81.4	0.168	14.8	0.136	2.6	0.111	28.0	0.150	5.7	0.060	18.4	0.569	4.3	0.796	2.0	0.236	0.4	0.279	3.5	0.132	2.5
	UDP	0.335	0.8	0.171	11.0	0.132	12.8	0.124	14.6	0.146	1.8	0.307	17.3	0.038	28.8	0.075	13.6	0.043	10.3	0.032	12.9	0.060	9.8	0.042	21.0	0.315	5.5	0.081	3.7	0.025	1.7	0.034	3.4	0.021	3.6
	UTP	0.276	7.1	0.205	16.6	0.174	24.7	0.190	29.3	0.193	5.1	0.339	11.9			0.078	18.4	0.041	10.4	0.044	58.0	0.052	7.7	0.020	25.1	0.197	3.6	0.390	1.0	0.128	1.3	0.175	0.6	0.105	1.9
	IMP	1.234	5.0	0.752	3.7	0.793	6.3	0.788	14.7	0.501	12.9	0.820	18.5	0.226	27.6	0.358	11.5	0.309	3.2	0.190	13.7	0.308	5.9	0.128	22.9	0.802	5.0	0.634	2.9	0.190	0.5	0.216	2.3	0.124	1.7
Deoxy nucleoside phosphates	dAMP	0.004	9.629	0.002	9.7	0.002	16.1	0.001	8.2	0.001	6.4	0.002	3.4	0.002	29.4	0.001	3.1	0.001	10.9	0.000	12.0	0.001	15.3	0.001	16.4	0.009	4.2	0.007	4.7	0.002	1.2	0.002	15.7	0.001	4.0
	dADP	0.037	7.274	0.020	13.1	0.021	33.9	0.033	45.5	0.014	6.4	0.026	15.2	0.008		0.014	14.1	0.012	6.7	0.011	68.1	0.010	3.1	0.003	20.8	0.180	2.9	0.486	4.2	0.098	8.0	0.139	3.5	0.063	2.0
	dATP	0.052	16.996	0.030	10.7	0.025	16.4	0.024	27.8	0.016	8.7	0.034	16.4	0.015	16.1	0.023	16.6	0.012	4.9	0.007	35.8	0.012	3.1	0.004	17.3	0.062	6.3	0.145	4.9	0.032	6.3	0.044	1.4	0.020	2.0
	dGMP	0.192	8.946	0.078	12.8	0.029	16.1	0.018	1.3	0.017	11.1	0.041	10.3	0.409	41.6	0.060	24.8	0.021	20.5	0.013	78.7	0.030	11.6	0.027	27.9	0.010	7.1	0.017	7.0	0.003	7.1	0.002	2.6	0.001	10.7
	dGDP	0.044	12.884	0.029	6.1	0.026	27.0	0.045	26.0	0.023	7.0	0.029	22.8	0.011	25.3	0.019	14.9	0.013	12.2	0.004	53.1	0.011	9.9	0.004	21.0	0.099	6.7	0.396	4.6	0.037	2.7	0.037	5.4	0.013	2.4
	dGTP	1.272	2.077	1.119	11.2	0.877	25.3	0.784	0.2	0.540	14.3	1.004	11.1	0.425	15.6	0.481	8.7	0.290	6.7	0.241	39.4	0.284	6.0	0.111	13.3	0.022	4.6	0.034	6.3	0.004	2.8	0.005	2.3	0.003	4.3
	dCTP	0.042	16.005	0.030	10.5	0.024	20.3	0.022	28.9	0.015	3.4	0.027	20.6	0.008	18.6	0.023	13.3	0.007	13.9	0.004	52.0	0.006	12.7	0.002	17.1	0.031	2.9	0.053	0.6	0.007	6.8	0.012	1.1	0.007	3.2
	dUMP	0.001	5.631	0.000	8.9	0.000	9.3	0.000	1.4	0.000	5.9	0.000	15.6	0.000	19.9	0.000	5.4	0.000	8.1	0.000	21.0	0.000	23.9	0.000	6.9	0.001	7.3	0.000	9.1	0.000	2.6	0.000	13.2	0.000	6.8
	dUTP	1.425	85.109	0.563	11.9	0.369	12.5	0.251	18.6	0.251	1.2	0.419	10.3	3.310	16.3	0.556	14.0	0.275	6.9	0.152	21.6	0.337	12.3	0.250	5.6	0.002	128.8	0.002	4.1	0.001	10.2	0.002	1.5	0.001	4.0
	dIMP	0.028	11.509	0.019	17.9	0.019	23.2	0.035	46.6	0.014	9.3	0.021	22.8	0.010	56.3	0.015	12.5	0.009	9.2	0.008	54.2	0.009	11.7	0.003	25.0	0.010	4.2	0.037	2.8	0.004	6.5	0.006	9.0	0.002	2.1
Other sugar phosphates	PRPP	0.061	16.607	0.068	16.9	0.042	46.2	0.046	53.1	0.056	5.9	0.081	12.6	0.009	58.0	0.053	25.1	0.012	24.2	0.066	72.8	0.011	17.4	0.006	12.3	0.171	3.0	0.124	2.0	0.007	1.4	0.014	3.4	0.011	4.1
	Gal1P	0.042	5.505	0.031	18.4	0.036	5.6	0.031	8.3	0.022	16.3	0.051	9.1			0.011	6.6	0.009	12.1	0.005	22.9	0.012	5.1	0.006	18.0	0.032	9.4	0.047	11.4	0.012	4.3	0.032	15.2	0.009	3.8
	G-/M1P	0.113	4.678	0.116	11.0	0.101	9.5	0.111	11.3	0.167	16.3	0.293	21.6	0.012	1.2	0.049	1.4	0.038	18.0	0.034	19.9	0.073	3.5	0.037	9.6	0.109	5.4	0.140	2.8	0.027	8.7	0.059	3.6	0.016	1.4
	F1P	0.032	16.451	0.030	20.0	0.019	29.6	0.015	56.6	0.042	8.4	0.065	15.9	0.008	35.2	0.021	8.7	0.007	29.2	0.006	40.9	0.014	8.5	0.006	10.2	0.008	6.4	0.033	41.4	0.009	8.5	0.016	7.6	0.007	1.3
	M6P	0.391	5.177	0.404	9.2	0.333	8.8	0.362	9.6	0.418	4.9	0.718	18.1	0.070	54.3	0.218	3.4	0.120	1.8	0.113	39.2	0.156	3.0	0.083	9.5	0.635	5.8	0.787	1.2	0.109	2.4	0.208	13.0	0.063	3.8
	GL3P	0.054	2.867	0.038	8.4	0.038	6.1	0.022	4.7	0.012	2.7	0.048	59.4	0.025	9.4	0.028	3.3	0.010	7.8	0.007	22.7	0.017	3.0	0.016	8.3	0.495	2.8	0.363	3.8	0.176	4.7	0.197	5.6	0.108	0.4
	UDP-glu	0.710	7.148	0.466	17.2	0.469	13.7	0.577	18.9	0.388	4.0	0.708	20.6	0.065	50.2	0.147	18.0	0.136	4.0	0.104	51.5	0.137	15.9	0.048	18.6	0.928	4.3	1.215	2.0	0.277	2.9	0.444	3.1	0.201	5.2
	UDP-Glc-Na	1.334	7.428	0.710	10.8	0.719	10.3	0.726	9.0	0.705	5.4	1.418	15.7	0.010	123.7	0.308	17.4	0.249	3.7	0.189	30.6	0.269	4.2	0.114	16.8	0.888	10.4	1.221	2.6	0.278	3.2	0.432	2.1	0.250	2.8
	DOXP	0.063	7.630	0.034	8.0	0.032	4.1	0.027	2.1	0.010	0.3	0.024	26.9			0.021	5.3	0.009	1.5	0.004	13.0	0.010	5.1	0.003	15.6	0.046	6.5	0.025	13.8	0.005	1.7	0.007	6.2	0.004	7.3
	2-IPML	0.027	4.097	0.023	9.5	0.015	3.8	0.021	6.0	0.010	2.0	0.050	63.9			0.014	5.7	0.005	2.7	0.015	18.1	0.027	5.1	0.002	20.1	0.030	3.9	0.018	0.6	0.003	0.2	0.013	2.4	0.004	3.9
	IPP	0.038	8.583	0.027	21.0	0.022	5.3	0.019	1.7	0.007	8.7	0.014	9.0			0.014	13.8	0.006	1.8	0.009	19.8	0.013	3.5	0.004	21.8	0.185	8.2	0.103	5.7	0.038	15.2	0.056	3.7	0.040	10.3
	GlcN6P	0.125	11.968	0.238	10.4	0.152	15.2	0.128	11.8	0.060	7.0	0.118	15.3	0.005	45.8	0.073	6.0	0.035	9.4	0.048	21.1	0.108	8.0	0.030	12.1	0.065	5.4	0.183	1.6	0.042	3.6	0.065	5.2	0.020	1.4
CoAs	CoA	0.026	11.492	0.041	64.2	0.094	36.3	0.086	1.4	0.052	56.5	0.053	12.1	0.069	15.4	0.088	36.5	0.101	11.7	0.123	5.8	0.088	14.6	0.043	18.3										
	AcCoA	0.204	32.081	0.079	32.0	0.280	23.7	0.129	3.4	0.136	55.0	0.202	11.4	0.157	20.3	0.115	32.0	0.138	11.6	0.060	2.7	0.028	13.6	0.007	18.4										
	SucCoA	0.006	27.909	0.006	25.7	0.017	7.7	0.024	10.4	0.022	23.3	0.023	11.7	0.045	13.3	0.053	23.1	0.077	19.3	0.180	9.7	0.119	13.5	0.051	15.1										
	MalCoA	0.003	19.823	0.007	45.2	0.004	28.5	0.007	6.6	0.006	59.4	0.008	13.0	0.002	20.7	0.003	34.3	0.004	20.5	0.009	13.2	0.009	11.5	0.004	8.6										
NADs	NAD	0.560	45.815	1.375	21.3	0.756	35.6	1.089	6.8	1.211	8.9	1.157	0	0.721	28.8	0.728	1.3	0.969	9.1	0.725	43.3	0.295	36.9	0.165	0.7										
	NADH	0.356	50.355	0.320	27.2	0.143	44.9	0.172	17.4	0.200	5.7	0.192	0	0.335	15.6	0.344	11.0	0.165	9.4	0.089	5.3	0.019	47.1	0.004	28.2										
	NADP	0.059	41.987	0.229	24.2	0.130	38.5	0.234	3.9	0.185	7.1	0.220	0	0.111	22.1	0.128	1.5	0.235	13.5	0.419	44.4	0.134	41.3	0.067	3.9										
	NADPH	0.027	12.352	0.203	35.5	0.057	7.5	0.040	8.4	0.106	46.1	0.147	0	0.173	83.8	0.033	39.6	0.152	101.1	0.065	35.8	0.058	28.6	0.016	51.4										
	FAD	0.095	37.571	0.153	21.6	0.094	38.1	0.129	5.4	0.116	5.7	0.125	0	0.084	24.0	0.121	3.0	0.156	7.6	0.242	39.4	0.096	37.6	0.044	1.8										
